# PSII Activity Was Inhibited at Flowering Stage with Developing Black Bracts of Oat

**DOI:** 10.3390/ijms22105258

**Published:** 2021-05-17

**Authors:** Bei Liu, Di Zhang, Ming Sun, Manli Li, Xiqing Ma, Shangang Jia, Peisheng Mao

**Affiliations:** 1 Forage Seed Laboratory, College of Grassland Science and Technology, China Agricultural University, Beijing 100193, China; liubei5191@163.com (B.L.); dichuengcau@163.com (D.Z.); sunming4709@163.com (M.S.); lmlcau@126.com (M.L.); ma2016@cau.edu.cn (X.M.); shangang.jia@cau.edu.cn (S.J.); 2 Key Laboratory of Pratacultural Science, Beijing Municipality, Yuanmingyuan West Road, Haidian District, Beijing 100193, China

**Keywords:** anthocyanin, chlorophyll, bract, flowering stage, oat, *p*-coumaric acid, photosynthesis

## Abstract

The color of bracts generally turns yellow or black from green during cereal grain development. However, the impact of these phenotypic changes on photosynthetic physiology during black bract formation remains unclear. Two oat cultivars (*Avena sativa* L.), ‘Triple Crown’ and ‘Qinghai 444’, with yellow and black bracts, respectively, were found to both have green bracts at the heading stage, but started to turn black at the flowering stage and become blackened at the milk stage for ‘Qinghai 444’. Their photosynthetic characteristics were analyzed and compared, and the key genes, proteins and regulatory pathways affecting photosynthetic physiology were determined in ‘Triple Crown’ and ‘Qinghai 444’ bracts. The results show that the actual PSII photochemical efficiency and PSII electron transfer rate of ‘Qinghai 444’ bracts had no significant changes at the heading and milk stages but decreased significantly (*p* < 0.05) at the flowering stage compared with ‘Triple Crown’. The chlorophyll content decreased, the LHCII involved in the assembly of supercomplexes in the thylakoid membrane was inhibited, and the expression of *Lhcb1* and *Lhcb5* was downregulated at the flowering stage. During this critical stage, the expression of *Bh4* and *C4H* was upregulated, and the biosynthetic pathway of *p*-coumaric acid using tyrosine and phenylalanine as precursors was also enhanced. Moreover, the key upregulated genes (*CHS*, *CHI* and *F3H*) of anthocyanin biosynthesis might complement the impaired PSII activity until recovered at the milk stage. These findings provide a new insight into how photosynthesis alters during the process of oat bract color transition to black.

## 1. Introduction

Bracts, the outer protective structure of flower and seed organs in Gramineae, consist of lemma and palea [1]. Most bracts contain chlorophyll and are photosynthetic tissues [2]. In recent years, the photosynthetic capacity of non-foliar green tissues has been extensively explored for its great significance in promoting crop growth and improving seed yield [3]. Previous studies in fruit and cereal crops have provided evidence demonstrating photosynthesis in non-foliar tissues as a potential target for further improvement to enhance crop yield [4]. As a non-foliar tissue in direct contact with the seed, bracts can provide material nutrition resulting from its photosynthesis to the growth of flower and seed. However, the molecular physiology of bract photosynthesis remains largely unknown [5,6,7].

Bract color is among the important morphological characteristics in Gramineae plants [8,9]. During the reproductive growth of cereal plants, bracts usually turn yellow from green at heading, flowering and seed maturation. They could also turn black, such as in rice (*Oryza sativa* L.) and oat (*Avena sativa* L.). The formation of black bracts is related to the accumulation of melanin. Melanin is a strong antioxidant, which can endow seeds with more vitality [10]. Melanin can enhance the mechanical strength of the bract, protecting the developing seeds [11]. It can also protect plants from excessive light damage and improve resistance to *Fusarium* spp. [12,13,14]. Varga et al. [15] found that the component of melanin in oat bracts was homopolymer of *p*-coumaric acid, one of the key intermediates in the anthocyanin biosynthesis pathway with phenylalanine as a precursor, which was catalyzed by cinnamate 4-hydroxylase (C4H) [16,17]. In wild rice, black is considered to be the original color in the bracts controlled by two complementary genes—*Bh4* (*black hull 4*) and *Phr1* (*phenol reaction 1*)—encoding a tyrosine transporter and a polyphenol oxidase, respectively, which are involved in melanin biosynthesis [18,19,20,21]. Notably, recent studies have shown that the formation of melanin in barley (*Hordeum vulgare* L.) bracts occurred in chloroplasts [22]. The development of bracts is accompanied by the formation of the black phenotype, but the variation of photosynthesis in this process has yet to be clarified and needs further research.

The thylakoid membrane complex system of photosynthetic organisms mainly includes photosystem II (PSII), photosystem I (PSI), cytochrome *b_6_f* (Cyt *b_6_f*) and ATP synthase (ATPase). PSII and PSI bind to different light harvesting pigment protein complexes (LHCs, including LHCII and LHCI) [23,24,25]. Generally, LHCI contains four proteins—Lhca1, Lhca2, Lhca3, Lhac4, with a molecular weight of 20–24 kDa—and could bind to chlorophyll a, chlorophyll b, zeaxanthin and *β*-carotene [26,27]. LHCII is a family of pigment protein complexes (i.e., Lhcb) with similar structure and evolutionary relationship, which are formed by proteins encoded by nuclear genes and pigments [28]. LHCII accounts for nearly 50% of the pigments in photosynthetic membrane and about 1/3 of the proteins [29]. At present, it is generally believed that LHCII contains six proteins, among which Lhcb1, Lhcb2 and Lhcb3 are the main light harvesting complexes, mostly in the form of trimers, while Lhab4, Lhcb5 and Lhcb6 are the secondary light harvesting complexes as monomers [30]. LHCs are capturing photons, driving photosynthetic electron transferring, generating oxygen from water, and converting NADP^+^ to NADPH, together with ATP production [31,32,33]. At the elongating, gelatinous, and early dough stages, the expression of photosynthesis-related genes such as those encoding LHCI, oxygen-evolving complex (OEC) and chlorophyll a/b binding protein in bracts was significantly higher than that in flag leaf [5]. However, the expression of the genes encoding PSII protein CP47, PSI protein PsaA and PsaB in rice bracts was significantly upregulated at the flowering stage and milk stage compared with that at the heading stage [34]. The development of bracts is closely related to the regulation of thylakoid membrane complex, but the molecular mechanism of bract color change and its regulation remain unclear.

Oat, an important annual cereal crop grown worldwide, can provide proteins, soluble fibers (β-glucans), unsaturated fatty acids, vitamins, minerals, and antioxidants [35,36]. It has become increasingly more attractive in recent years due to the discovery of its many health benefits such as lowering cholesterol and reducing glycemic response [37]. In 2017, the global output of oats attained 26 million tons, and this is expected to increase further in the future [38]. Bract color is one of the most important characters in variety identification, and yellow bracts are commonly seen in oat cultivars, such as ‘Triple Crown’ and ‘Qingyin No 1’, while black bracts are found in other cultivars, including ‘Qinghai 444’ and ‘Qingyan No 1’ [39,40]. During the growth and development of oat, the physiological change rule and the molecular regulation mechanism of photosynthesis in the process of color change in bracts from green to yellow or black are still unclear. To this end, we have selected two oat cultivars, ‘Triple Crown’ and ‘Qinghai 444’, to analyze and compare the photosynthetic characteristics of bracts and the changes of thylakoid membrane complexes in chloroplasts at different developmental stages, and explore the key proteins of the photosynthetic regulation pathway in the formation of black bracts. The results obtained provide a theoretical basis for deciphering the physiological and molecular regulation mechanism in photosynthesis during oat maturation.

## 2. Results

### 2.1. Changes of Color and Photosynthetic Pigment Content during the Oat Bract Development

**Figure 1:**
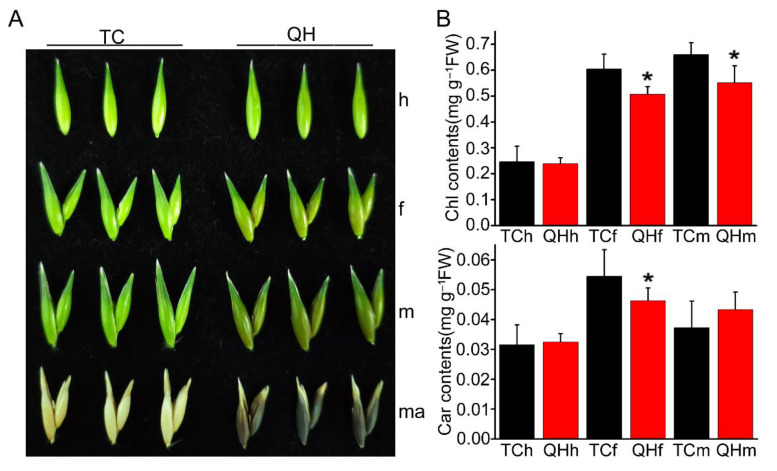
Phenotype identification and photosynthetic pigment content measurement during oat bract development. (**A**) Identification of the phenotype of bracts; TC indicates ‘Triple Crown’; QH indicates ‘Qinghai 444’; h, f, m and ma represent the heading, flowering, milk, and mature stages, respectively. (**B**) The content of photosynthetic pigments in bracts was determined. Bracts at different stages were selected, and the total chlorophyll content and carotenoid content were measured. The asterisk represents a significant difference in total chlorophyll and carotenoid content of ‘Triple Crown’ and ‘Qinghai 444’ bracts (* *p* ˂ 0.05, Student’s *t*-test), with 6 replicates.

Two cultivars, ‘Triple Crown’ and ‘Qinghai 444’, show different colors in bracts from heading to the mature seed stage (Figure 1A). The bracts of ‘Triple Crown’ and ‘Qinghai 444’ were both green at the heading stage, but changed their color at the flowering stage. The green bracts of ‘Triple Crown’ turned yellow with the development from the flowering to the mature seed stage, while those of ‘Qinghai 444’ turned black, exhibiting a special phenotype for oat grain.

Furthermore, the photosynthetic pigment content of bracts at the heading, flowering and milk stages were measured for ‘Triple Crown’ and ‘Qinghai 444’ (Figure 1B). The results showed that there was no significant difference between ‘Triple Crown’ and ‘Qinghai 444’ for the total chlorophyll and carotenoid contents in bracts at the heading stage. However, compared with ‘Triple Crown’, the total chlorophyll content of ‘Qinghai 444’ decreased significantly (*p* < 0.05) at the flowering and milk stage, while carotenoids only decreased significantly (*p* < 0.05) at the flowering stage. These data indicated that the total chlorophyll and carotenoid contents in bracts of ‘Qinghai 444’ were lower than ‘Triple Crown’, with bracts color gradually turning dark at flowering stage.

### 2.2. Changes of Photosynthetic Physiological Characteristics during Oat Bract Development

**Figure 2:**
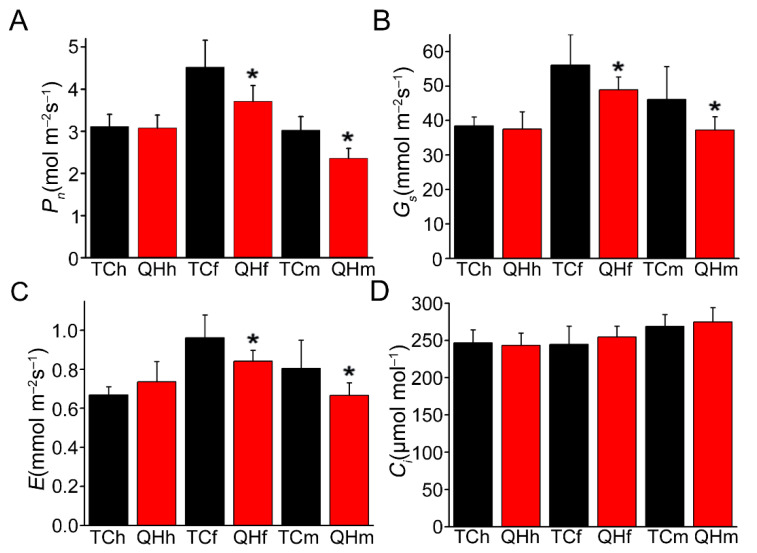
Gas exchange parameters of bracts during oat bract development. (**A**) *P_n_*, the net photosynthetic rate; (**B**) *G_s_*, stomatal conductance; (**C**) *E*, the transpiration rate; (**D**) *C_i_*, the intercellular carbon dioxide concentration. The asterisk represents significant difference in ‘Triple Crown’ and ‘Qinghai 444′ bracts (* *p* ˂ 0.05, Student’s *t*-test), with 9 replicates.

Photosynthetic parameters including net photosynthetic rate (*P_n_*), transpiration rate (*E*), stomatal conductance (*G_s_*) and substomatal CO_2_ concentrations (*C_i_*) were measured for the oat bracts during development, from the heading to milk stages, and significant differences were observed between ‘Triple Crown’ and ‘Qinghai 444’ (Figure 2). Although, there were no significant differences (*p* > 0.05) for *P_n_*, *E* and *G_s_* in the bracts at the heading stage between ‘Triple Crown’ and ‘Qinghai 444’. *P_n_*, *E* and *G_s_* of ‘Qinghai 444’ bracts were significantly (*p* < 0.05) lower than those of ‘Triple Crown’ at the flowering and milk stages (Figure 2A–C). Furthermore, there were no significant differences for *C_i_* in the bracts between ‘Triple Crown’ and ‘Qinghai 444’ during development from the heading to milk stages (Figure 2D).

**Figure 3:**
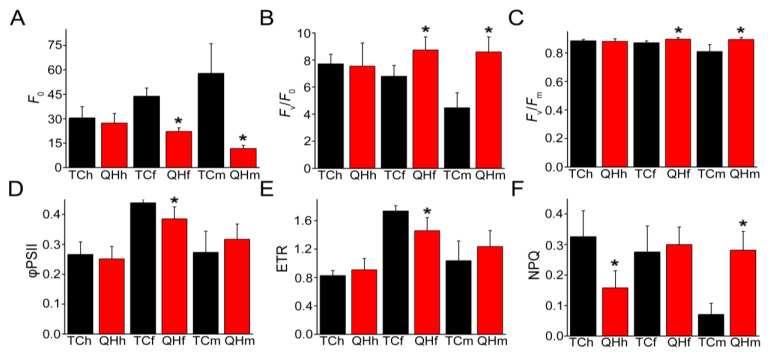
Chlorophyll fluorescence parameters of bracts during oat bract development. (**A**) *F*_0_, the minimum fluorescence; (**B**) *F*_v_/*F*_0_, PSII potential photosynthetic activity; (**C**) *F*_v_/*F*_m_, PSII maximum photochemical efficiency; (**D**) φPSII, actual PSII photochemical efficiency; (**E**) ETR, PSII electron transfer rate; (**F**) NPQ, non-photochemical quenching. The asterisk represents the difference in ‘Triple Crown’ and ‘Qinghai 444’ bracts (* *p* ˂ 0.05, Student’s *t*-test), with 3 replicates.

The measurement of chlorophyll fluorescence in the bract developing from the heading to milk stages revealed three types of changing trends between ‘Triple Crown’ and ‘Qinghai 444’ in some parameters, including non-photochemical quenching (NPQ), minimum fluorescence (*F*_0_), actual PSII photochemical efficiency (φPSII), PSII electron transfer rate (ETR), PSII potential photosynthetic activity (*F*_v_/*F*_0_), and PSII maximum photochemical efficiency (*F*_v_/*F*_m_) (Figure 3). The changes of *F*_0_, *F*_v_/*F*_0_ and *F*_v_/*F*_m_ were similar during the development between ‘Triple Crown’ and ‘Qinghai 444’; φPSII and ETR also exhibited a similar trend (Figure 3A–E). At the heading stage, the NPQ in ‘Qinghai 444’ bracts was the only parameter significantly (*p* < 0.05) lower than that in ‘Triple Crown’ (Figure 3F). At the flowering stage, *F*_0_, φPSII and ETR in ‘Qinghai 444’ were significantly (*p* < 0.05) higher than those in ‘Triple Crown’, but *F*_v_/*F*_0,_ and *F*_v_/*F*_m_ were significantly (*p* < 0.05) lower (Figure 3A–E). Up to the milk stage, only *F*_0_ in ‘Qinghai 444’ maintained a significantly (*p* < 0.05) lower level, whereas *F*_v_/*F*_0_, *F*_v_/*F*_m_ and NPQ in ‘Qinghai 444’ were all significantly (*p* < 0.05) higher than those in ‘Triple Crown’ (Figure 3A–C,F).

### 2.3. Transcriptome Analysis and Comparison for the Oat Bracts at the Different Developing Stages

**Figure 4:**
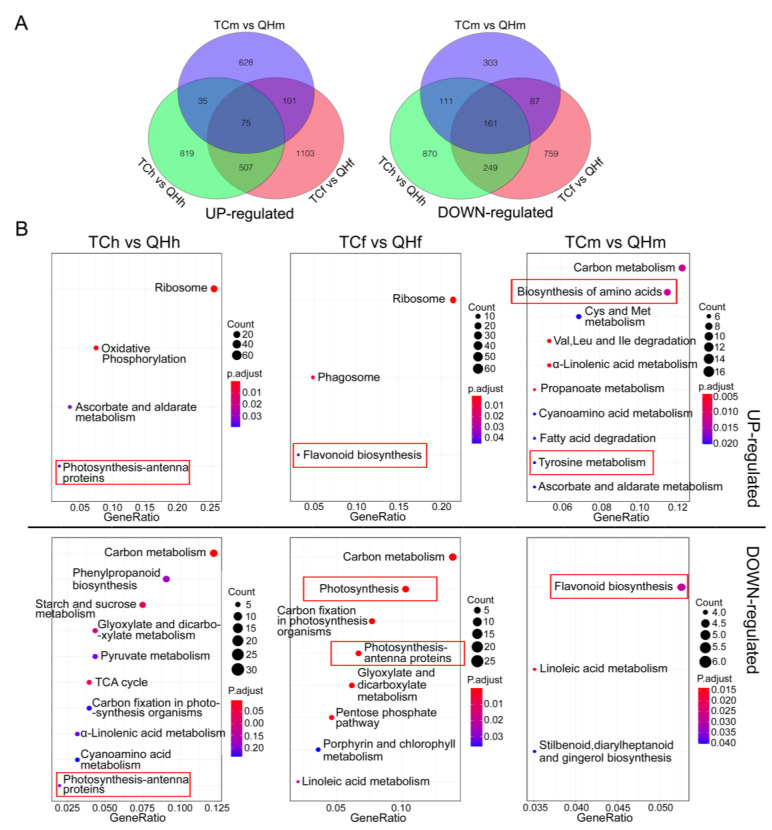
The enrichments of DEGs during oat bract development. (**A**) Venn diagrams of upregulated and downregulated DEGs in ‘Qinghai 444’, compared to those in ‘Triple Crown’. (**B**) KEGG enrichment of upregulated and downregulated DEGs in ‘Qinghai 444’, compared to those in ‘Triple Crown’. Red square line represents the pathways which are related to photosynthesis and anthocyanin or melanin biosynthesis in this study.

Analysis of the transcriptome sequence and data comparison between the cultivars of ‘Triple Crown’ and ‘Qinghai 444’ were carried out for the bracts sampled at the heading, flowering and milk stages. The results of principal component analysis showed that there were differences in gene expression of ‘Triple Crown’ vs. ‘Qinghai 444’ in the three stages, and a significant difference in gene expression occurred in the flowering stage, i.e., TCf (‘Triple Crown’ in the flowering stage) vs. QHf (’Qinghai 444’ in the flowering stage) (Figure S1). Data analysis of the transcriptome for ‘Triple Crown’ vs. ‘Qinghai 444’ in the three stages identified a total of 5656 DEGs, of which 3268 were upregulated while 2540 were downregulated (Figure 4A). The upregulated DEGs consisted of 1436 DEGs in the heading, 1786 DEGs in the flowering and 839 DEGs in the milk stage, and the downregulated DEGs consisted of 1396 DEGs in the heading, 1256 DEGs in the flowering and 662 DEGs in the milk stage.

KEGG enrichment analysis revealed a difference in the metabolism pathways in bracts in the different developing stages between ‘Qinghai 444’ and ‘Triple Crown’ (Figure 4B). Ribosome and oxidative phosphorylation pathways were upregulated, whereas carbon metabolism and phenylpropanoid biosynthesis pathways were downregulated, and all these pathways in ‘Qinghai 444’ bracts were enriched in the heading stage. In particular, the upregulated and downregulated photosynthesis-antenna proteins were enriched in the TCh (‘Triple Crown’ in the heading stage) vs. QHh (‘Qinghai 444’ in the heading stage). Ribosome, phagosome and flavonoid biosynthesis pathways were upregulated, whereas carbon metabolism, photosynthesis and carbon fixation in photosynthesis organism pathways were downregulated, and all these pathways in ‘Qinghai 444’ bracts were enriched in the flowering stage. In particular, downregulated photosynthesis-antenna proteins were only enriched in the TCf vs. QHf. Carbon metabolism, the biosynthesis of amino acids and tyrosine metabolism pathways were upregulated, flavonoid biosynthesis and linoleic acid metabolism pathways were downregulated, and all these pathways in ‘Qinghai 444’ bracts were enriched in the milk stage. Upregulated flavonoid biosynthesis was especially enriched in the TCf vs. QHf, but was downregulated in the TCm (‘Triple Crown’ in the milk stage) vs. QHm (‘Qinghai 444’ in the milk stage).

GO enrichment analysis (Figure S2) revealed that ribosome and chloroplast stroma processes were upregulated; hydrolase activity, hydrolyzing *O*-glycosyl compounds and cell wall were downregulated; and all these processes in ‘Qinghai 444’ bracts were enriched in the heading stage. In particular, the upregulated and downregulated chloroplast thylakoid membrane were both enriched in the TCh vs. QHh. Ribosome and cytosolic large ribosomal subunit processes were upregulated; chloroplast thylakoid membrane, photosynthesis, photosystem I and II processes were downregulated; and all these processes in ‘Qinghai 444’ bracts were enriched in the flowering stage. Only the downregulated chloroplast thylakoid membranes were both enriched in the TCf vs. QHf. Nucleosome and protein heterodimerization activity processes were upregulated; oxylipin biosynthetic protein folding was downregulated; and all these processes in ‘Qinghai 444’ bracts were enriched in the milk stage. Similarly, the upregulated and downregulated chloroplast thylakoid membranes were both enriched in the TCm vs. QHm.

**Figure 5:**
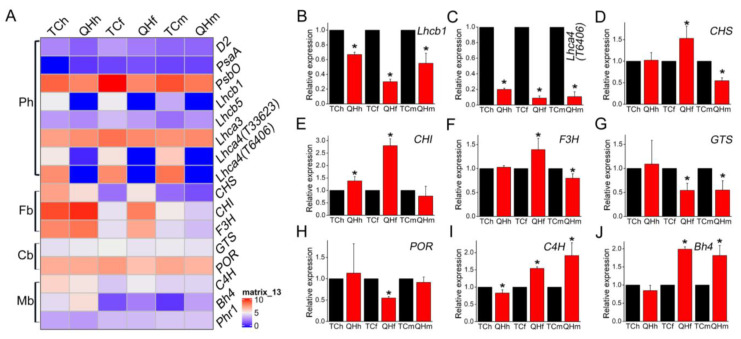
Heatmap and qRT-PCR verification of key genes during oat bract development. (**A**) Heat map analysis of the expression of key genes at the three stages. “Ph”, photosynthesis; “Fb”, flavonoid biosynthesis; “Cb”, chlorophyll biosynthesis; “Mb”, melanin biosynthesis. (**B**–**J**) qRT-PCR analysis of key genes at the three stages. Relative expression values of ‘Qinghai 444’ samples were calculated and normalized with ‘Triple Crown’ as the control in one single stage. The asterisk represents significant differences in relative expression of genes (* *p*˂0.05, Student’s *t*-test), with 3 replicates for each experiment.

Some key genes in photosynthesis, flavonoid biosynthesis, chlorophyll biosynthesis, and melanin biosynthesis were selected for further analysis by qRT-PCR (Figure 5). It was found that *Lhca4* (gene ID: *avena_sativa_T6406* and *avena_sativa_T33623*) of LHCI and *Lhcb1* of LHCII were significantly (*p* < 0.05) downregulated in all three stages of ‘Qinghai 444’. *Lhca3* of LHCI was significantly (*p* < 0.05) downregulated in the flowering stage of ‘Qinghai 444’, whereas *Lhcb5* of LHCII exhibited no significant differences in all three stages. Additionally, oxygen-evolving complex gene *PsbO* in PSII was significantly (*p* < 0.05) downregulated at the flowering and milk stages (Figure 5A and Figure S3C). Furthermore, *Lhca4* (gene ID: *avena_sativa_T6406*) with the fold change ranked the second in the list of top 50 downregulated DEGs between ‘Qinghai 444′ and ‘Triple Crown’. *Lhca4* (gene ID: *avena_sativa_T33623*) was ranked as fifteenth at the flowering stage and fourteenth at the milk stage, respectively. *Lhcb1* was found at the flowering stage as the twentieth (Table S2).

Analysis of the key genes involved in chlorophyll biosynthesis showed that *glutamyl-tRNA synthetase* (*GTS*) and *chlorophyllide reductase* (*POR*) were significantly (*p* < 0.05) downregulated at the flowering stage of ‘Qinghai 444’ bracts compared with ‘Triple Crown’ (Figure 5A,H,I).

Analysis of the key genes involved in anthocyanin biosynthesis showed that *chalcone* *synthase* (*CHS*), *chalcone isomerase* (*CHI*) and *flavanone3-hydroxylase* (*F3H*) were all significantly (*p* < 0.05) upregulated at the flowering stage of ‘Qinghai 444’ bracts compared with ‘Triple Crown’. However, the expression of *CHS* and *F3H* was significantly (*p* < 0.05) downregulated at the milk stage for ‘Qinghai 444’ bracts (Figure 5A,D–F and Figure S3F).

Expression analysis of the genes related to melanin synthesis showed that while *C4H* in ‘Qinghai 444’ bracts was significantly (*p* < 0.05) upregulated at the flowering stage (Figure 5A,I), the expression of *Bh4* in ‘Qinghai 444’ was significantly (*p* < 0.05) upregulated at the flowering and milk stages (Figure 5A,J). However, no significant (*p* > 0.05) difference in *Phr1* expression was observed between ‘Triple Crown’ and ‘Qinghai 444’ (Figure 5A).

### 2.4. Photosynthetic Membrane Complexes and Its Subunits for the Oat Bracts in the Different Developing Stages

**Figure 6:**
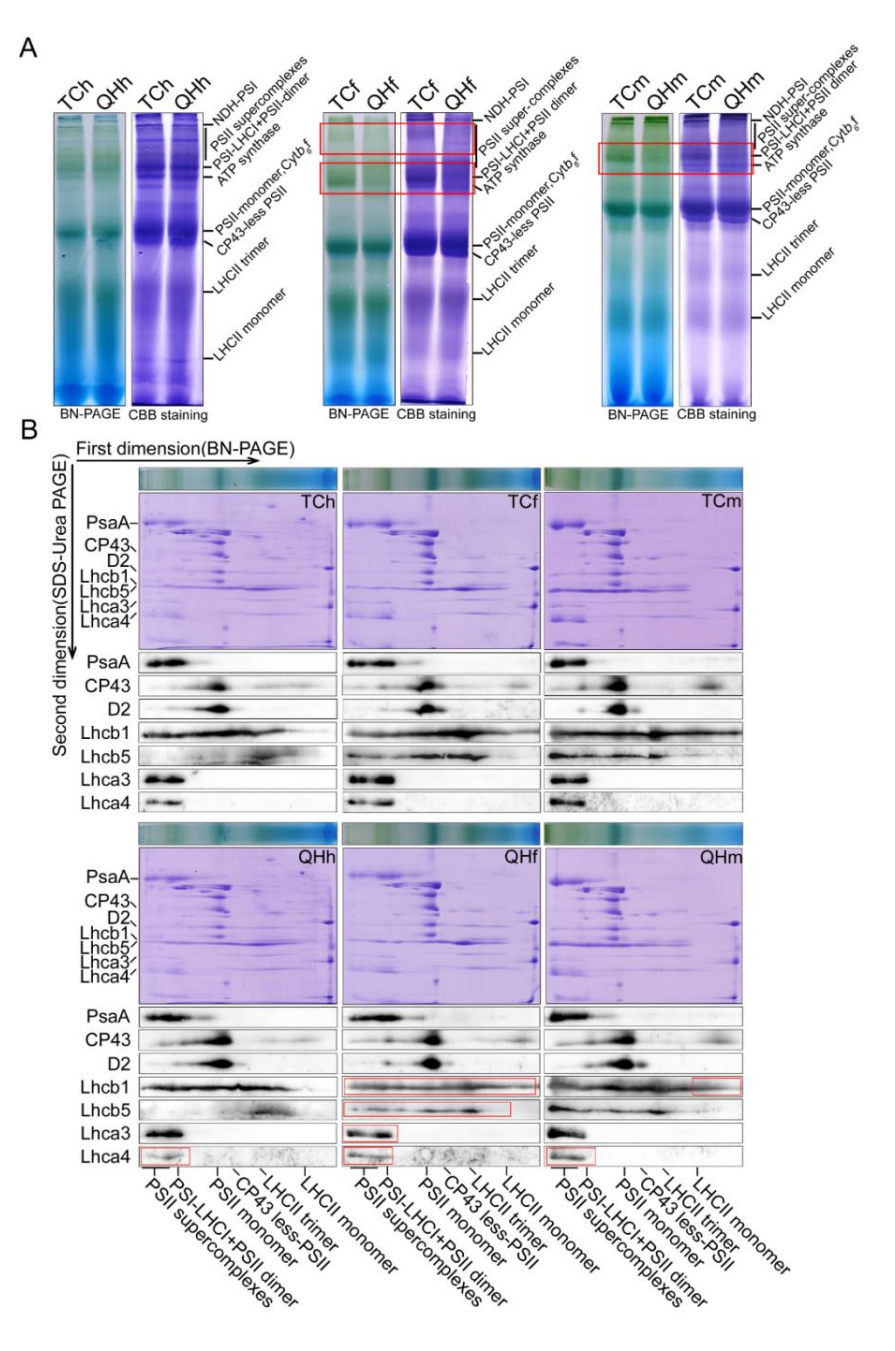
Accumulation of thylakoid membrane protein complexes and their subunits during oat bract development. (**A**) BN-PAGE analysis of thylakoid membrane protein complexes. The thylakoid membrane was solubilized with 1% DM, and the total protein was loaded (100 µg). The thylakoid membrane protein complex was separated in 5–12% BN-gel, and then, CBB stained (right). (**B**) BN/SDS-PAGE two-direction electrophoresis analysis of thylakoid membrane protein complexes. After the thylakoid membrane protein complex was separated in the first direction by BN-PAGE, it was further separated in the second direction by 15% SDS-urea-PAGE, and then, stained with CBB and Western blotting. Red square line represents significantly different complexes or subunit proteins.

In order to explore the role of the photosynthetic membrane complex in the bracts with three stages, BN-PAGE and its two-dimensional electrophoresis experiments were conducted. The results showed that the PSI–LHCI complex and the PSII supercomplex contents in ‘Qinghai 444’ both clearly decreased at the flowering and milk stages, and at the flowering stage, respectively (Figure 6A). The Western blotting results of two-dimensional electrophoresis showed that the contents of PsaA in PSI and D2, and CP43 in PSII had no differences between ‘Qinghai 444’ and ‘Triple Crown’ bracts. Furthermore, the content of Lhca4, the constituent subunits of LHCI, declined in ‘Qinghai 444’ at the heading, flowering and milk stages, while the content of Lhca3, the constituent subunits of LHCI, Lhcb1 and Lhcb5 in LHCII decreased at the flowering stage in both cultivars (Figure 6B). Western blotting using specific antibodies revealed no obvious differences for the accumulation of photosynthesis-related proteins in the heading, flowering and milk stages between ‘Qinghai 444’ and ‘Triple Crown’ bracts (Figure S4).

## 3. Discussion

Bracts including palea and lemma are a unique flower organ in Gramineae plants. It is important to study the development and molecular regulation mechanism of flower organs to improve seed yield and provide new quality traits [41,42]. While previous research on bract development has mostly been focused on the gene regulation of morphological and structural changes, few studies have been focused on the color changes of the bracts [1,43,44]. During their development in ‘Triple Crown’ and ‘Qinghai 444’, the green bracts gradually turned yellow and black from the heading, flowering, milk to the mature stage, and the difference in bract color phenotype between the two oat cultivars began to appear at the flowering stage. Moreover, the color change in bracts affected the normal photosynthesis process in ‘Qinghai 444’, and was associated with changes in chlorophyll, anthocyanin, melanin and other components.

### 3.1. Color Change in Bracts and Photosynthetic Physiological Parameters

With the accumulation of melanin in bracts of ‘Qinghai 444’, the relative expression of key enzyme genes *GTS* and *POR* decreased in the chlorophyll biosynthesis pathway, and resulted in the inhibition of chlorophyll synthesis (Figure 1A and Figure 5G,H). A decrease in chlorophyll content would lead to a reduction in light energy absorption efficiency, and inhibition of plant photosynthesis. *P_n_* was a direct indicator of photosynthetic capacity, which was also affected by *G_s_*, *C_i_*, *E* and chlorophyll fluorescence [45]. In this study, the gas exchange parameters, *P_n_*, *G_s_* and *E* all decreased significantly (*p* < 0.05), although *C_i_* increased with no significant difference, indicating that the decrease in *P_n_* might be caused by non-stomatal factors. Weng et al. [46] proved that the variation of φPSII and ETR in flag leaves of rice was consistent with *P_n_* during development from the heading to the mature grain stage. Varone et al. [47] found that the decrease in ETR in leaves of three Mediterranean plants at the seedling stage had an impact on *P_n_*. The chlorophyll fluorescence parameters measurement in ‘Qinghai 444’ bracts showed that there was no significant difference for *F*_0_, *F*_v_/*F*_0_, *F*_v_/*F*_m_, ETR, and φPSII, and only NPQ decreased significantly (*p* < 0.05) at the heading stage (Figure 3). However, with the changing of bract color at the flowering stage, the chlorophyll content, ETR and φPSII of ‘Qinghai 444’ all decreased significantly (*p* < 0.05), indicating a loss of PSII activity (Figure 3D,E). This phenomenon was also illustrated with the decreasing relative expression of PSII gene *D2* (Figure S3A). Wang et al. [48] found that under drought stress in wheat (*Triticum aestivum* L.), an increase in *D2* transcription level could enhance PSII activity and promote photosynthesis. In addition, the downregulated expression of oxygen-evolving complex gene *PsbO* in ‘Qinghai 444’ corresponded to the decrease in *P_n_* (Figure S3C). Li and Yi [49] found that *P_n_* increased and *PsbO* transcription was upregulated with SO_2_ treatment in *Arabidopsis thaliana*.

### 3.2. Candidate Gene Bh4 for Oat Black Bracts

**Figure 7:**
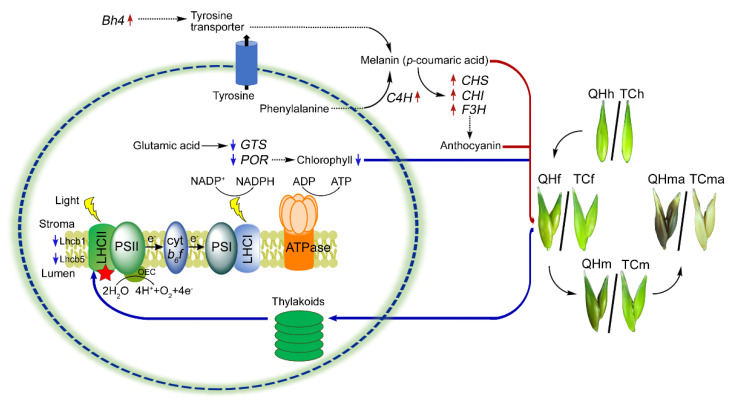
A molecular regulatory scheme of photosynthesis in response to color changes at the flowering stage. The dotted line indicates that the process is unknown, or some steps are omitted. The red and blue arrows indicate up- and downregulation, respectively. Red stars indicate LHCII which are impaired at flowering stage.

Little is known about the role of the regulating genes that determine the black phenotype in oat bracts. The melanin in oat bracts was proved to be a homopolymer of *p*-coumaric acid, and *C4H* encodes a key enzyme for *p*-coumaric acid synthesis [15,16]. The transcription of *C4H* in ‘Qinghai 444’ bracts was significantly (*p* < 0.05) upregulated at the flowering and milk stages (Figure 5I). This indicated that the enhanced activity of key enzymes in melanin biosynthesis could cause the accumulation of melanin, consistent with the observed color change of bracts. Meanwhile, for the regulation of melanin biosynthesis, the expression of candidate genes, *Bh4* and *Phr1*, were compared, and the relative expression of the *Bh4* homologous gene (Figure 5J), rather than that of *Phr1*, was found to be significantly (*p* < 0.05) increased at the flowering and milk stages in ‘Qinghai 444’ (Figure 5A). Hence, it could be speculated that the biosynthesis of melanin in oat bract might be regulated by the *Bh4* gene (Figure 7).

### 3.3. Complementation of Anthocyanin for PSII Activity

The KEGG analysis of DEGs showed that the expression of genes related to the flavonoid biosynthesis pathway was significantly (*p* < 0.05) upregulated at the flowering stage in ‘Qinghai 444’ compared to ‘Triple Crown’ (Figure 4B). Similarly, the relative expression levels of *CHS*, *CHI* and *F3H*, the key genes in anthocyanin biosynthesis, were also significantly (*p* < 0.05) upregulated (Figure 5A,D–F). The formation of melanin was accompanied by the enhanced expression of key genes in anthocyanin biosynthesis and declined accumulation of carotenoid at the flowering stage (Figure 1B). The anthocyanins accumulation of ‘Qinghai 444’ at the flowering stage might enhance the heat dissipation capacity, eliminating the excess light energy to restore PSII potential activity to a certain extent, and increase the value of *F*_v_/*F*_0_ and *F*_v_/*F*_m_. Anthocyanins played important roles in alleviating PSII damage, affecting oxygen-evolving complex activity [50,51,52], and improving the light protection of leaves under UV wavelength [53]. Compared with ‘Triple Crown’, the relative expression of key genes of the anthocyanin synthesis pathway in ‘Qinghai 444’ bracts was significantly (*p* < 0.05) downregulated at the milk stage, while ETR, φPSII went back to a normal level without significant differences (Figure 5A,D–F and Figure 3D,E). A newly published research study showed that plants with high photosynthetic performance had higher NPQ enhancement ability [54]. The increase in NPQ, *F*_v_/*F*_0_ and *F*_v_/*F*_m_ suggested a restoration of PSII activity [55].

*p*-coumaric acid was found to have cross action between melanin and anthocyanin biosynthesis in oat bracts. Tyrosine was one of the precursors in *p*-coumaric acid biosynthesis [56]. In KEGG analysis, upregulated DEGs in the tyrosine metabolism pathway were significantly (*p* < 0.05) enriched at the milk stage (Figure 4B), suggesting its close relationship with melanin synthesis. In Arabidopsis, tyrosine could indirectly mediate the expression of late anthocyanin biosynthesis genes [20]. In addition, phenylalanine was another precursor in *p*-coumaric acid biosynthesis, and p-coumaric acid was also a key intermediate for anthocyanin biosynthesis [17]. There was a balance in the accumulation of *p*-coumaric acid and anthocyanin, as the complementary effects for gene expressions at the flowering and milk stages were observed (Figure 7).

### 3.4. LHCII Involved in the Assembly of Supercomplexes Was Inhibited

BN-PAGE was a powerful technology to study the thylakoid membrane and other cell membrane systems [57,58]. In this study, it was confirmed by the BN-PAGE result that the contents of the PSI–LHCI and the PSII supercomplex in ‘Qinghai 444’ bracts decreased at the flowering stage (Figure 6). However, the subunit contents of D2, CP43 in PSII and PsaA in PSI had little change in all three stages (Figure 6B). *F*_0_ was the fluorescence of chlorophyll and was excited by light harvesting antenna pigment; a decrease in *F*_0_ value indicated a loss of photosynthetic pigment [59,60]. *F*_0_ in ‘Qinghai 444’ bracts decreased significantly (*p* < 0.05) at the flowering and milk stages, indicating a decreasing pigment content in light harvesting antenna complexes. Meanwhile, the LHC genes, including *Lhcb1*, *Lhcb5*, *Lhca3* and *Lhca4*, exhibited a significant decrease in transcription at the flowering stage (Figure 4B and Table S2). These BN-PAGE results were consistent with transcriptome analysis and qPCR verification, indicating that only LHCs, especially LHCII in photosynthesis, were sensitive to the changes in bract color.

Western blotting showed that the accumulation of 12 selected subunits, including Lhcb1 and Lhca4 in mainly thylakoid membrane complexes, was at the same level in the three different stages (Figure S4). The results showed that the change in bract color had no effect on the accumulation of photosynthesis-related proteins, but it might have a direct effect on the content of light harvesting antenna protein subunits in the assembly process of the supercomplex. Therefore, the PSI–LHCI and PSII supercomplex content could be decreased at the flowering stage.

## 4. Materials and Methods

### 4.1. Plant Materials and Growth Conditions

The seeds of ‘Triple Crown’ (TC) and ‘Qinghai 444’ (QH) used in the experiments were from the Forage Seed Laboratory of China Agricultural University, and harvested (20 kg) at Yuershan farm in Hebei Province, China (41°44′ N, 116°8′ E; 1455 m elevation), in 2018. The bract color of ‘Triple Crown’ and ‘Qinghai 444’ was yellow and black, respectively, at the mature stage. The seeds were planted in an artificial climate chamber (20 °C, 70% humidity, 1200 μmol m^−2^ s^−1^, 16 h light and 8 h dark) in September 2019. The oat seedling facility consisted of a substrate ratio of Pindstrup substrate and vermiculite equal to 2:1 in a plastic flowerpot of 23 cm height and 16cm width; four oat seeds were randomly planted in each pot. Bract color was observed at the heading stage (TCh, QHh), flowering stage (TCf, QHf), milk stage (TCm, QHm) and mature stage (TCma, QHma) in both ‘Triple Crown’ and ‘Qinghai 444’; 4 g oat bracts at the heading, flowering and milk stages were cut for subsequent experiments.

### 4.2. Photosynthetic Pigment Content Measurements

One fresh intact oat bract was collected at different developmental stages with three random repetitions in three plastic flowerpots, weighed and cut into filaments before being transferred to a 1.5 mL plastic tube. After an addition of 0.6 mL 80% acetone, the tube was incubated overnight at room temperature (22 °C) in the dark until oat bracts turned completely white. The absorbance values at 663 nm, 645 nm and 470 nm were measured with 80% acetone as the blank sample. The concentrations of chlorophyll and carotenoid were calculated using the following formula [61]:

Chlorophyll concentration = Chlorophyll a + Chlorophyll b = (12.21 × OD633-2.81 × OD646) + (20.13 × OD646-5.03 × OD663) = 7.18 × OD633 + 17.32 × OD646

Carotenoids concentration = (1000 × OD470 − 3.27 × Ca − 104 × Cb)/229

Photosynthetic pigment content (mg/g) = C (mg/L) × total amount of extract (mL)/weight of bracts (g) × 1000

### 4.3. Gas Exchange Measurements

The gas exchange in oat bract at different developmental stages was measured according to Jin et al. [62]. The net photosynthetic rate (*P_n_*), stomatal conductance (*G_s_*), transpiration rate (*E*) and intercellular CO_2_ concentrations (*C_i_*) of intact bracts were measured using a CIRAS-3 portable photosynthesis system (PP Systems, Amesbury, MA, USA) at the conditions of 25 °C, 1200 μmol m^−2^ s^−1^ PPFD, 50–60% relative humidity and 380 μmol mol^−1^ CO_2_. The well-developed bracts of three oat spikelets in a plastic pot were randomly selected and placed in the PLC-3 (7 × 25 mm) leaf chamber for determination, and then the data were recorded once. The whole measurement process was recorded three times, with a total of nine repeats.

### 4.4. Chlorophyll Fluorescence Measurements

The method described by Wingler et al. [63] was adopted for measuring chlorophyll fluorescence parameters in oat bract at different developmental stages using an FMS-2 pulse modulation fluorometer (Hansatech, UK). After 15 min of dark adaptation, minimum fluorescence yield (*F*_0_) was recorded by measuring light, and maximum fluorescence yield (*F*_m_) was recorded with a saturating flash, while variable fluorescence yield (*F*_v_) was calculated under dark adaptation. After 15 min of illumination, maximum fluorescence of light-adapted leaves (*F*_m_^’^), steady-state fluorescence (*F*_s_) and ground fluorescence (*F*_0_^’^) were recorded under light adaption. The following equations were used for calculating photosynthetic parameters.

Maximum photochemical rate, *F*_v_/*F*_m_ = (*F*_m_ − *F*_0_)/*F*_m_*;* PSⅡ potential photochemical activity *F*_v_/*F*_0_; actual photochemical efficiency, φPSII = (*F*_m_^′^ − *F*_s_)/*F*_m_^′^; non-photochemical quenching NPQ = (*F*_m_ − *F*_m_^′^)/*F*_m_^′^; photosystem II electron transfer rate, ETR = φPSII × 0.84 × 0.5.

### 4.5. Thylakoid Membrane Fractionation

The method described by Li et al. [64] was used for thylakoid membrane fractionation in oat bracts at different developmental stages. Fresh bracts (4 g) were collected in the tubes with three replicates. After addition of 20 mL medium I (0.33 M sorbitol, 20 mM tricine/KOH pH 8.4, 5 mM EGTA pH 8.35, 5 mM EDTA pH 8.0, 10 mM NaHCO_3_), the sample was filtered into a three-layer Miracloth. The pellets were resuspended three times in 1 mL medium II (0.33 M sorbitol, 5 mM MgCl_2_, 2.5 M EDTA pH 8.0, 20 mM HEPES/KOH pH 7.6) after centrifuging at 4 °C and 4200× *g* for 5min; in 1mL medium III (5 mM MgCl_2_, 2.5 M EDTA pH 8.0, 20 mM hepes/KOH pH 7.6) after centrifuging at 4 °C and 8000× *g* for 2 min; and in 500 μL medium III after centrifuging at 4 °C and 8000× *g* for 2 min. The total thylakoid membrane protein was quantified with a Pierce^®^ BCA Protein Assay Kit (Thermo, NCI3227CH).

### 4.6. Electrophoresis and Immunoblotting

A slightly modified method by Peng et al. [65] was used for BN-PAGE in oat bracts at different developmental stages. The isolated thylakoid membranes from fresh bracts (4× *g*) were gently washed twice with buffer containing 25 mM Bis-Tris/HCl (pH 7.0) and 20% glycerol, and were solubilized in buffer containing 25 mM Bis-Tris/HCl (pH 7.0), 20% glycerol, and 1% DM, at a final protein concentration of 10 mg/mL. After incubation on ice for 30 min and centrifugation at 12,000× *g* for another 10 min, the supernatants were supplemented with 1/10 volume of BN sample buffer (30% glycerol, 5% Serva blue G-250, 0.5 M 6-aminocaproic acid, 100 mM Bis-Tris/HCl, pH 7.0). Thylakoid protein complexes were separated by 5–12% gradient BN-PAGE in 0.75-mm thick gels. For two dimensional SDS-urea-PAGE/Western blotting analysis, excised BN-PAGE lanes were soaked in SDS sample buffer (50 mM Tris-HCl, pH 6.8, 5% SDS, 20% glycerol, 8 M urea, 5%-mercaptoethanol) for 30 min at room temperature and then layered onto 1-mm thick 15% gels.

For normal immunoblotting, thylakoid membranes were resuspended in SDS sample buffer containing 1% bromophenol blue. Protein samples were separated by SDS-urea-PAGE using 15% (*w*/*v*) acrylamide gels containing 6M urea and transferred to nitrocellulose membranes. Proteins were bound with twelve specific antibodies belonging to each thylakoid membrane complex (PSII-D2, CP43; PSI-PsaA, PsaD; ATPase-CF_1_α; Cyt *b_6_f*-Cyt *f*, Cyt *b_6_*; OEC-PsbO; LHCII-Lhcb1, Lhcb5; LHCI-Lhca3, Lhca4) in TTBS buffer (20 mM Tris-HCl, pH7.4, 150 mM NaCl) with 1% skimmed milk, and immunoblot signals were measured by the chemiluminescence method in an AlphaImager HP multifunctional imaging analysis system, FluorChemR (Protein Sample, Santa Clara County, CA, USA).

### 4.7. Transcriptome Sequencing and Analysis

Total RNA was extracted from fresh intact oat bracts at different developmental stages according to the manufacturer’s instructions for TRIzol reagent (Invitrogen, Carlsbad, CA, USA). RNA concentration was measured using a NanoDrop 2000 (Thermo Fisher, Waltham, MA, USA). RNA integrity was assessed using the RNA Nano 6000 Assay Kit of the Agilent Bioanalyzer 2100 system (Agilent Technologies, CA, USA). A total amount of 1μg RNA per sample with three replicates was used for library construction using the NEBNext^®^Ultra™ RNA Library Prep Kit for Illumina^®^ (NEB, Ipswich, MA, USA). Briefly, mRNA was purified from total RNA using poly-T oligo-attached magnetic beads. First strand cDNA was synthesized using random hexamer primer and M-MuLV Reverse Transcriptase. Second strand cDNA synthesis was subsequently performed using DNA Polymerase I and RNase H. The cDNAs were fragmented for a preferred size of 240 bp, and the library preparations were sequenced on an Illumina Hiseq 2000 platform with paired-end mode.

We created an oat transcriptome reference (unpublished) for RNA-seq analysis in this study. Full-length transcriptome sequencing was performed based on an RNA pool of embryos, endosperm, seedlings, leaves, stems, roots and florets. After de-redundancy, 67,184 high-quality transcript sequences were obtained. RNA-seq data were analyzed with trimming by Trimmomatic-0.39 [66], mapping by TopHat2 [67] on the above oat transcriptome reference, and mRNA expression values were determined by HTSeq [68]. R functions prcomp() and ggbiplot() were employed for principal component analysis (PCA) and visualization. Significantly differentially expressed genes (DEGs) were identified by the R package DESeq2 [69], according to fold change ≥ 2.0 and the adjusted *p* value ≤ 0.05. GO and KEGG enrichment analyses on the DEGs were performed in R package clusterProfiler [70]. The RNA-seq raw data were deposited in the Short Reads Archive (SRA) of NCBI (accession No: PRJNA728512).

### 4.8. Quantitative RT-PCR Analysis

All gene-specific primers used in qRT-PCR experiments were designed by Primer Premier 5 [71] (Table S2). The RNA was extracted from each sample, and reverse transcribed into cDNA by using the PrimeScript^TM^RT reagent kit (RR047A, TAKARA, Japan). cDNA was diluted 10-fold for qRT-PCR analysis. Each sample was amplified three times using SYBR Premix Ex Taq (Takara, Japan) on the Bio-Rad CFX96 real-time PCR detection system (Bio-rad, Hercules, CA, USA), with *Actin-2* as the internal control (Table S1). The relative quantification (2^−ΔΔCT^) of target gene expression was calculated using the comparative cycle threshold method [72].

## 5. Conclusions

Based on the above results, we mapped a molecular regulatory scheme of photosynthesis in response to color changes during oat bract development. (Figure 7). The φPSII and ETR of ‘Qinghai 444’ bracts had no significant change at the heading and milk stages, but decreased significantly (*p* < 0.05) at the flowering stage compared with ‘Triple Crown’ and the bract color began to change at this time. The chlorophyll content decreased, LHCII involved in the assembly of supercomplexes in the thylakoid membrane was inhibited, and the expression of *Lhcb1* and *Lhcb5* was downregulated at the flowering stage. During this critical stage, the expression of *Bh4* and *C4H* was upregulated, and the biosynthetic pathway of *p*-coumaric acid using tyrosine and phenylalanine as precursors was enhanced. The key genes of anthocyanin biosynthesis (*CHS, CHI* and *F3H*) were also upregulated, indicating their role in complementation for PSII activity. Then, the activity of PSII could be recovered to the original level at the milk stage. However, the mechanism of gene regulating remains unclear for the transition from green to black in bract color change during oat development. The regulatory network among the key genes of melanin, anthocyanin and chlorophyll biosynthesis and the subunit genes of the light harvesting antenna complex needs to be studied further. These results provided a foundation for better understanding of the molecular mechanism of photosynthesis limitation at the flowering stage during the development of black bract, and also established a theoretical basis for using genetic modification to improve the photosynthetic potential of non-foliar tissues.

## Supplementary Materials

The following are available online at https://www.mdpi.com/article/10.3390/ijms22105258/s1.
