# Supplementary material for: PSII Activity Was Inhibited at Flowering Stage with Developing Black Bracts of Oat
Source: Int J Mol Sci. 2021 May 17;22(10):5258. doi: 10.3390/ijms22105258 (PMC8156022; doi:10.3390/ijms22105258)
Supplement: Supplementary file 1 [file ijms-22-05258-s001.zip › ijms-1202607-supplementary.pdf]

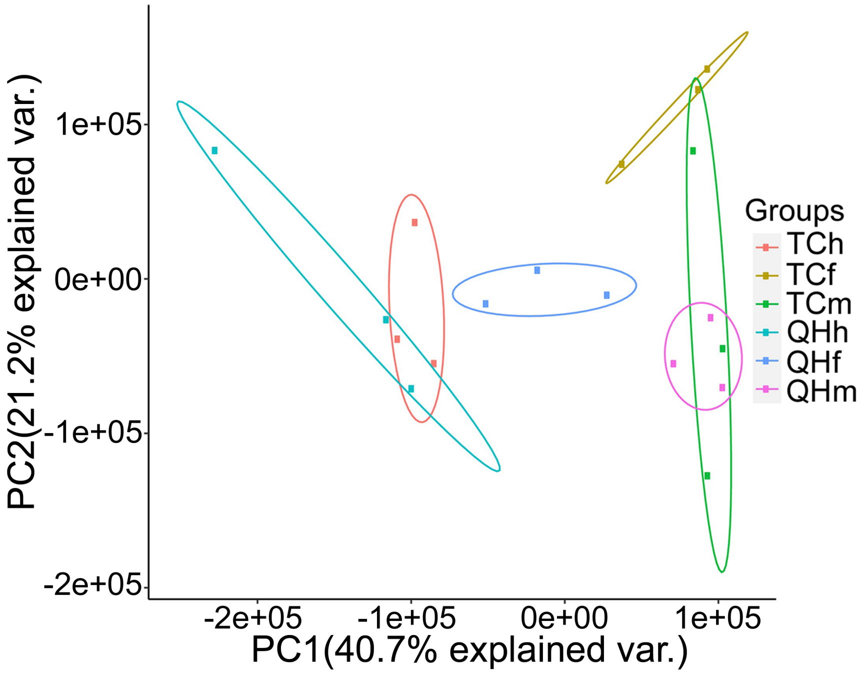

Supplementary Figure 1. Principal component analysis (PCA) during the oat bracts development. PCA plot for all the RNA-seq samples. Color points represent the sample replicates.

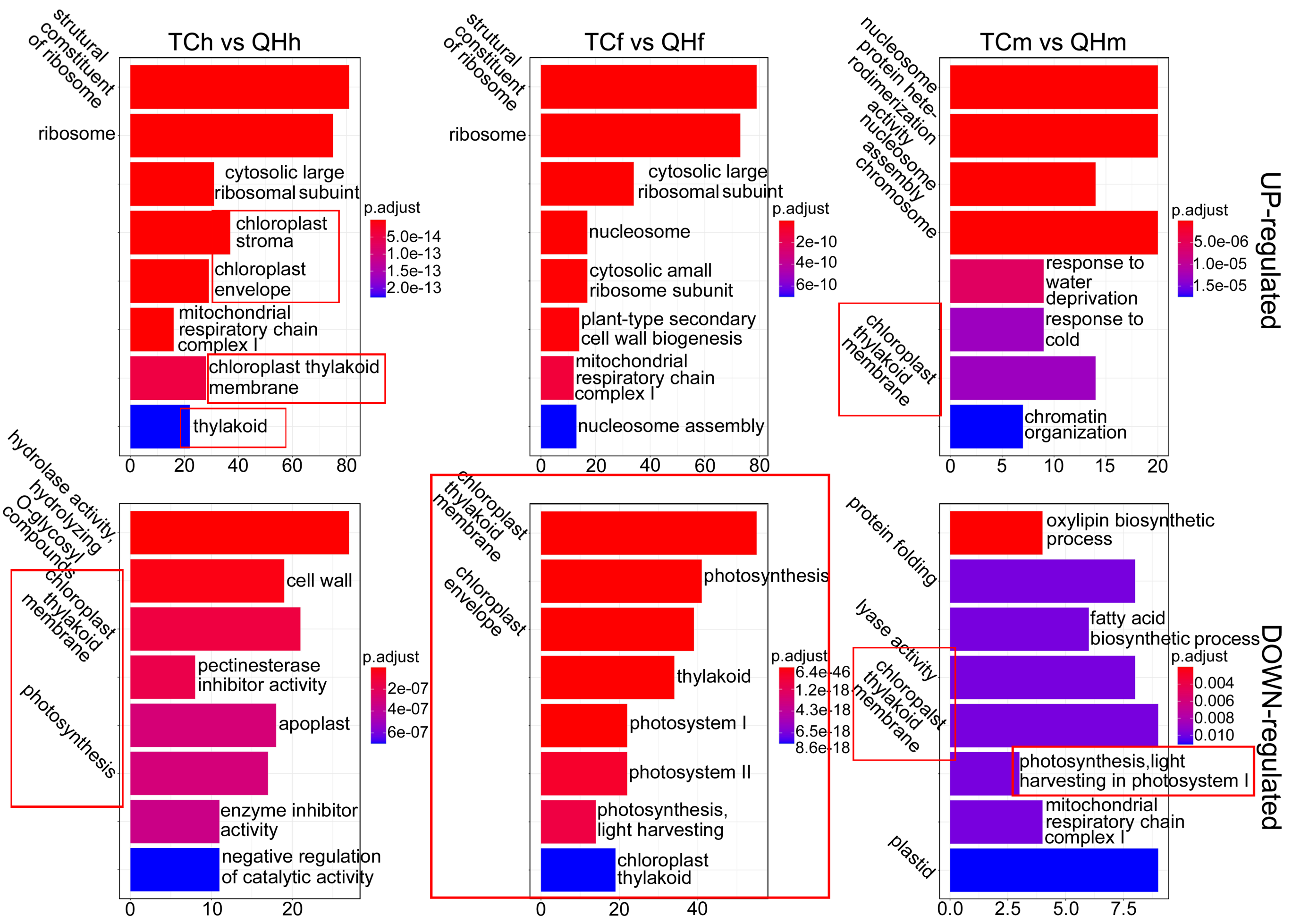

Supplementary Figure 2. Go enrichment analysis during the oat bracts development. Gene Ontology (GO) enrichment analysis of up-regulated and down-regulated DEPs in two oat cultivars at three stages. Red square line represents the processes which are related to photosynthesis in this study.

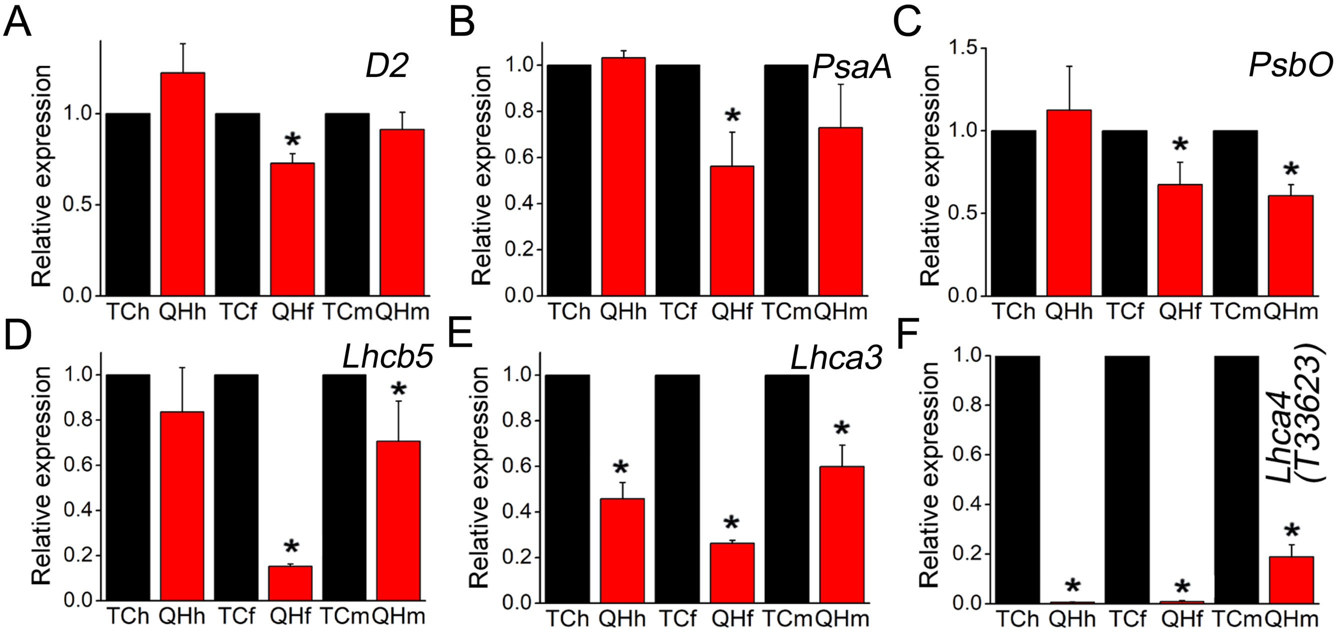

Supplementary Figure 3. qPCR verification of key genes during the oat bracts development. (A-F) relative expressions of genes of D2, PsaA, PsbO, Lhcb5, Lhca3 and Lhca4 in QH were calculated with TC samples as the control with three replicates.

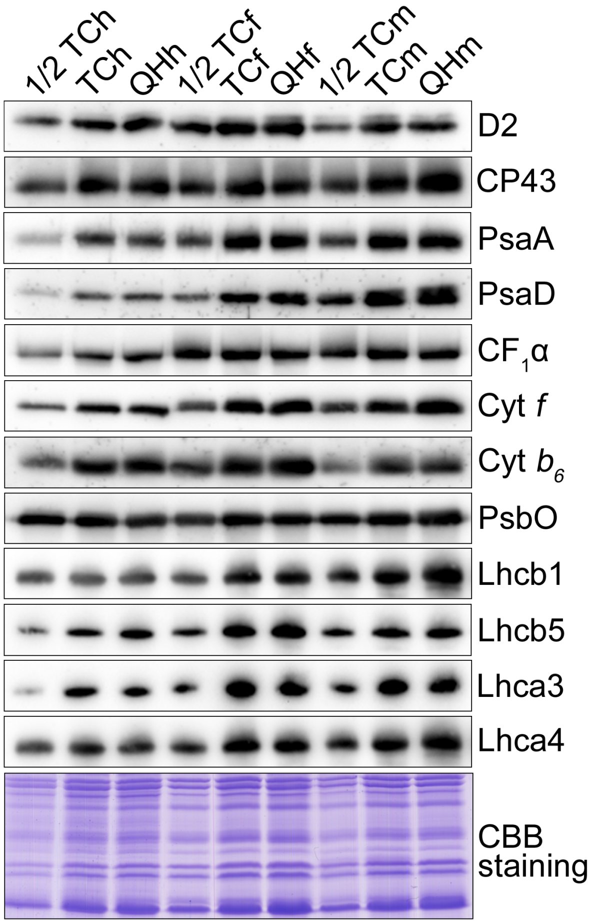

Supplementary Figure 4. Western blotting analysis during the oat bracts development. The intact thylakoid membrane proteins were extracted from bracts, and the whole proteins were loaded on 15% SDS-urea-PAGE for separation. And then the antibodies were used for western blotting analysis.

ID	Primer	Sequence(5'to3')
1	Actin2-F	GCTATTCAAGCCGTGCTTTC
2	Actin2-R	AGCATGTGGAAGGGCATAAC
3	D2-F	TTTTGTGTTCGGGAAAGTCC
4	D2-R	AGAGGTAGCAATTCGGAACG
5	PsaA-F	ACTTGCCTCGCATTGACTTC
6	PsaA-R	ATTTCTGCCGCTTCTTCTCC
7	Lhca4(T6406)-F	TCTGTCCACTCGGGAAACAT
8	Lhca4(T6406)-R	CACCACTGCACCCTTTAGGT
9	Lhca4(T33623)-F	TTAGCTCTGGCCTTTTTGGA
10	Lhca4(T33623)-R	CACCACTGCACCCTTTAGGT
11	Lhca3(T39441)-F	TCTGAAGGCATCTTCGTCCT
12	Lhca3(T39441)-R	ACCCACCCTCGCACATATAC
13	Lhcb1(T6439)-F	TCCCAGACTTTGGAGAGCAT
14	Lhcb1(T6439)-R	TGCTTTCTTGAGACACCAAATG
15	Lhcb5(T5352)-F	CATCCTCCCTTCTTGCTGAG
16	Lhcb5(T5352)-R	TGCTCAGGCAGCATAAGAAA
17	PsbO(T48477)-F	CAGACCCAACACACCAGACA
18	PsbO(T48477)-R	CAAAGAAGTTGGAGGCGAAC
19	CHS(T3168)-F	GCTGTCTCTTGCTTGCCTCT
20	CHS(T3168)-R	CACCTCACAACCCATTTTATGA
21	C4H(T2126)-F	AACAACCAACCGCTCGTTAC
22	C4H(T2126)-R	ACTATGCAGGAGGGCGTAGA
23	CHI(T7367)-F	CCATATACCATGGCCAGCTC
24	CHI(T7367)-R	TGACCTGGATGCAAGTGAAA
25	F3H(T57376)-F	GGTGCTTTTCTCCACTCTGG
26	F3H(T57376)-R	GCTTGCCCTGATCCATACAT
27	GTS(T1571)-F	TCCTCGTCGTAATCCAATCC
28	GTS(T1571)-R	CCAGCAGCGGAAGAAATAGA
29	PCE(T3550)-F	TATCTTGCTCGCTGGTTCAA
30	PCE(T3550)-R	CTGCGTCTGAATTTGCTGAA
31	Bh4(T2428)-F	AGAGGGAGGAGTGCGGATA
32	Bh4(T2428)-R	GTCCCAATGCATTCTTCTCC

Supplementary Table 1. Primers used in this study.

Top 50 up-regulated DEGs at heading stage(TCh vs QHh)

Gene ID	L3a.1	L3a.2	L3a.3	L4a.1	L4a.2	L4a.3	L3a_L4a_fc	L3a_L4a_fc2	L3a_L4a_pvalue	L3a_L4a_padj	KEGG	Annotation	Challenger Gene ID	oat-ot3098-pepsico*
avena_sativa_T48798	0	0	0	49	57	46	0.000328839	-11.5703301	2.34E-10	7.66E-08	K02969|8.09706e-70|bdi:100838410|K02969 small subunit ribosomal protein S20e | (RefSeq) 40S ribosomal protein S20	PREDICTED: 40S ribosomal protein S20 [Brachypo	avena_sativa_T4879	Pepsico1_Contig2227.path2
avena_sativa_T63067	0	0	0	44	44	34	0.000409668	-11.25325658	2.32E-09	0.000000652	K10405|0|bdi:100834158|K10405 kinesin family member C1 | (RefSeq) kinesin-5-like	PREDICTED: kinesin-5-like [Brachypodium distach	avena_sativa_T63067	TRINITY_DN15321_c0_g1_i6.path2
avena_sativa_T8917	0	0	0	5	9	106	0.000416493	-11.22941969	0.0000253	0.00182776	--	predicted protein [Hordeum vulgare subsp. vulgare]	avena_sativa_T8917	Pepsico1_Contig303.path1
avena_sativa_T2746	0	0	0	25	29	35	0.000561482	-10.7984718	4.37E-08	0.0000085	--	predicted protein [Hordeum vulgare subsp. vulgare]	avena_sativa_T2746	Pepsico2_Contig9991.path1
avena_sativa_T8833	0	0	0	3	4	79	0.000581058	-10.74903138	0.000257938	0.010764722	--	predicted protein [Hordeum vulgare subsp. vulgare]	avena_sativa_T8833	Pepsico1_Contig1710.path1
avena_sativa_T10796	0	0	0	33	24	25	0.000609385	-10.68035952	0.000000102	0.0000173	--	PREDICTED: cytochrome b5-like [Oryza brachyantl	avena_sativa_T1079	Pepsico2_Contig20097.path1
avena_sativa_T4152	0	0	0	23	25	21	0.000724113	-10.4314976	0.000000391	0.0000565	NA		avena_sativa_T4152	Pepsico2_Contig7629.path1
avena_sativa_T23950	0	0	0	20	20	27	0.000745712	-10.38909352	0.000000576	0.0000796	K04078|6.05959e-144|bdi:100821312|K04078 chaperonin GroES | (RefSeq) 20 kDa chaperonin	PREDICTED: 20 kDa chaperonin， chloroplastic-lil	avena_sativa_T2395	Pepsico1_Contig6962.path1
avena_sativa_T14113	0	0	0	1	1	58	0.000832639	-10.23002044	0.003334039	0.067900105	--	RecName: Full=Protein RAFTIN 1A; Short=TaRAF	avena_sativa_T14113	TRINITY_DN91660_c0_g1_i1.path1
avena_sativa_T60907	0	0	0	21	22	14	0.000876424	-10.15608308	0.000003	0.00032778	NA		avena_sativa_T60907	TRINITY_DN21683_c0_g2_i1.path1
avena_sativa_T9287	0	0	0	3	4	44	0.000979432	-9.995767151	0.000912266	0.028083036	--	PREDICTED: putative cell wall protein [Brachypodi	avena_sativa_T9287	Pepsico1_Contig1034.path1
avena_sativa_T10620	0	0	0	14	13	23	0.000999001	-9.967226259	0.0000101	0.00086924	--	Os06g0124900 [Oryza sativa Japonica Group]	avena_sativa_T1062	TRINITY_DN5740_c0_g1_i1.path1
avena_sativa_T48021	0	0	0	17	18	14	0.001019368	-9.938109326	0.00000783	0.000715671	NA		avena_sativa_T48021	Pepsico2_Contig3717.path1
avena_sativa_T19793	0	0	0	14	15	19	0.001040583	-9.908392621	0.00000934	0.000817696	K17991|1.81208e-130|bdi:100840663|K17991 peroxygenase [EC:1.11.2.3] | (RefSeq) probable peroxygenase 5	PREDICTED: probable peroxygenase 5 [Brachypodi	avena_sativa_T19793	Pepsico1_Contig112.path1
avena_sativa_T21612	0	0	0	21	21	5	0.001062699	-9.878050913	0.000089	0.004784272	--	cell wall-associated hydrolase [Phaseolus vulgaris]	avena_sativa_T21612	Pepsico2_Contig17422.path5
avena_sativa_T26571	0	0	0	11	12	22	0.001109878	-9.815383296	0.0000285	0.001967563	--	Transmembrane 9 superfamily member 4 [Aegilops t	avena_sativa_T26571	TRINITY_DN206_c0_g3_i2.path3
avena_sativa_T9658	0	0	0	15	15	15	0.001109878	-9.815383296	0.0000138	0.001118547	NA		avena_sativa_T9658	Pepsico2_Contig12156.path1
avena_sativa_T49767	0	0	0	2	4	36	0.001189061	-9.71596199	0.002051819	0.04894716	--	predicted protein [Hordeum vulgare subsp. vulgare]	avena_sativa_T49767	Pepsico1_Contig1034.path1
avena_sativa_T11023	0	0	0	17	23	1	0.001218027	-9.681238412	0.00142673	0.038045368	--	Major Cab protein [Petunia x hybrida]	avena_sativa_T11023	Pepsico1_Contig8041.path1
avena_sativa_T52192	0	0	0	14	14	13	0.001218027	-9.681238412	0.0000292	0.002001344	--	predicted protein [Hordeum vulgare subsp. vulgare]	avena_sativa_T52192	Pepsico2_Contig11530.path1
avena_sativa_T44083	0	0	0	14	12	9	0.001426534	-9.453270634	0.000117678	0.005982771	--	PREDICTED: uncharacterized protein LOC1039405	avena_sativa_T44083	NA
avena_sativa_T1241	0	0	0	3	4	25	0.001560062	-9.324180547	0.002699486	NA	--	predicted protein [Hordeum vulgare subsp. vulgare]	avena_sativa_T1241	Pepsico1_Contig21590.path2
avena_sativa_T59572	0	0	0	12	12	7	0.001610306	-9.278449458	0.000308596	NA	NA		avena_sativa_T59572	TRINITY_DN15509_c1_g1_i2.path1
avena_sativa_T16332	0	0	0	9	9	11	0.00172117	-9.182394353	0.000363575	NA	--	PREDICTED: reticulon-like protein B21 [Brachypod	avena_sativa_T16332	Pepsico1_Contig26214.path2
avena_sativa_T56339	0	0	0	6	8	15	0.00172117	-9.182394353	0.000667585	NA	--	hypothetical protein TRIUR3_05404 [Triticum urartu	avena_sativa_T5633	NA
avena_sativa_T1353	0	0	0	14	13	0	0.001848429	-9.079484784	0.02561537	NA	K08245|0|bdi:100839510|K08245 phytepsin [EC:3.4.23.40] | (RefSeq) aspartic proteinase	Aspartic proteinase [Triticum urartu]	avena_sativa_T1353	Pepsico1_Contig21373.path1
avena_sativa_T14859	0	0	0	4	7	15	0.001919386	-9.025139562	0.001874661	NA	NA		avena_sativa_T1485	Pepsico1_Contig20732.path1
avena_sativa_T9865	0	0	0	11	11	4	0.001919386	-9.025139562	0.001438568	NA	--	PREDICTED: uncharacterized protein LOC1008410	avena_sativa_T9865	Pepsico2_Contig3428.path1
avena_sativa_T10995	0	0	0	9	15	1	0.001996008	-8.968666793	0.006971564	NA	NA		avena_sativa_T1099	Pepsico2_Contig17422.path4
avena_sativa_T16395	0	0	0	8	9	8	0.001996008	-8.968666793	0.000912144	NA	K20367|1.03695e-110|bdi:100843594|K20367 endoplasmic reticulum-Golgi intermediate compartment protein 3 | (RefSeq) endoplasmic reticulum-Golgi intermediate compartment protein 3-like	PREDICTED: endoplasmic reticulum-Golgi interme	avena_sativa_T1639	Pepsico2_Contig10785.path2
avena_sativa_T58097	0	0	0	8	9	8	0.001996008	-8.968666793	0.000912144	NA	K00864|0|bdi:100831505|K00864 glycerol kinase [EC:2.7.1.30] | (RefSeq) glycerol kinase	glycerol kinase [Triticum aestivum]	avena_sativa_T5809	Pepsico1_Contig30909.path1
avena_sativa_T12851	0	0	0	7	7	10	0.002079002	-8.909893084	0.001278611	NA	--	PREDICTED: glutamate receptor 3.1-like [Brachypo	avena_sativa_T12851	Pepsico1_Contig35576.path1
avena_sativa_T61809	0	0	0	8	9	6	0.002169197	-8.84862294	0.001638644	NA	K10999|0|sita:101768666|K10999 cellulose synthase A [EC:2.4.1.12] | (RefSeq) probable cellulose synthase A catalytic subunit 1 [UDP-forming]	PREDICTED: probable cellulose synthase A catalyti	avena_sativa_T6180	Pepsico2_Contig21010.path2
avena_sativa_T23198	0	0	0	8	8	6	0.002267574	-8.784634846	0.002025692	NA	K13343|3.72796e-16|bdi:100825820|K13343 peroxin-14 | (RefSeq) peroxisomal membrane protein PEX14	Peroxisomal membrane protein PEX14 [Triticum ura	avena_sativa_T2319	TRINITY_DN43498_c0_g1_i1.path1
avena_sativa_T33770	0	0	0	5	5	11	0.002375297	-8.717676423	0.00372899	NA	K00012|0|bdi:100840467|K00012 UDPglucose 6-dehydrogenase [EC:1.1.1.22] | (RefSeq) UDP-glucose 6-dehydrogenase 4-like	PREDICTED: UDP-glucose 6-dehydrogenase 4-like	avena_sativa_T3377	Pepsico2_Contig12020.path1
avena_sativa_T3861	0	0	0	1	1	19	0.002375297	-8.717676423	0.028702431	NA	--	unnamed protein product [Triticum aestivum]	avena_sativa_T3861	Pepsico2_Contig808.path1
avena_sativa_T55671	0	0	0	6	6	9	0.002375297	-8.717676423	0.002762168	NA	K03255|0|bdi:100830075|K03255 protein TIF31 | (RefSeq) protein TSS	hypothetical protein F775_09910 [Aegilops tauschii]	avena_sativa_T55671	Pepsico2_Contig16552.path1

avena_sativa_T16032	0	0	0	1	2	17	0.002493766	-8.647458426	0.02386601	NA	--	unnamed protein product [Triticum aestivum]	avena_sativa_T16032	Pepsico2_Contig808.path1
avena_sativa_T28329	0	0	0	7	8	5	0.002493766	-8.647458426	0.003567746	NA	--	hypothetical protein F775_29651 [Aegilops tauschii]	avena_sativa_T28329	Pepsico2_Contig910.path1
avena_sativa_T42692	0	0	0	7	6	7	0.002493766	-8.647458426	0.003245313	NA	--	putative protein kinase [Oryza sativa Japonica Group	avena_sativa_T42692	TRINITY_DN8399_c1_g2_i1.path1
avena_sativa_T46781	0	0	0	7	7	5	0.002624672	-8.573647187	0.004451871	NA	--	OSJNBa0087O24.13 [Oryza sativa Japonica Group]	avena_sativa_T46781	Pepsico2_Contig20979.path1
avena_sativa_T55327	0	0	0	1	2	16	0.002624672	-8.573647187	0.026319105	NA	K15398|0|sita:101786122|K15398 fatty acid omega-hydroxylase [EC:1.14.-.-] | (RefSeq) cytochrome P450 86A4-like	predicted protein [Hordeum vulgare subsp. vulgare]	avena_sativa_T55327	Pepsico2_Contig2387.path1
avena_sativa_T57887	0	0	0	9	8	2	0.002624672	-8.573647187	0.008948278	NA	NA		avena_sativa_T57887	Pepsico1_Contig1970.path1
avena_sativa_T10100	0	0	0	4	4	10	0.002770083	-8.495855027	0.008442965	NA	K03938|1.16837e-33|sbi:SORBI_07g024460|K03938 NADH dehydrogenase (ubiquinone) Fe-S protein 5 | (RefSeq) SORBIDRAFT_07g024460	Sb07g024460; hypothetical protein， TRIUR3_2113	avena_sativa_T10100	Pepsico1_Contig2461.path3
avena_sativa_T17415	0	0	0	7	10	1	0.002770083	-8.495855027	0.017264053	NA	NA		avena_sativa_T17415	Pepsico2_Contig17636.path1
avena_sativa_T19571	0	0	0	6	6	6	0.002770083	-8.495855027	0.005458936	NA	--	PREDICTED: zinc finger BED domain-containing pı	avena_sativa_T19571	TRINITY_DN12832_c0_g2_i2.path1
avena_sativa_T11678	0	0	0	6	5	6	0.002932551	-8.413627929	0.007293922	NA	K15289|0|bdi:100830691|K15289 solute carrier family 35	member F5 | PREDICTED: uncharacterized transpoı	avena_sativa_T11678	Pepsico2_Contig19494.path1
avena_sativa_T39710	0	0	0	2	3	12	0.002932551	-8.413627929	0.018957145	NA	NA		avena_sativa_T39710	TRINITY_DN37953_c0_g1_i2.path1
avena_sativa_T47075	0	0	0	1	1	15	0.002932551	-8.413627929	0.042132279	NA	--	unnamed protein product [Triticum aestivum]	avena_sativa_T47075	Pepsico1_Contig5512.path1
avena_sativa_T46476	0	0	0	5	6	5	0.003115265	-8.326429487	0.009609434	NA	--	Os01g0630700 [Oryza sativa Japonica Group]	avena_sativa_T46476	Pepsico2_Contig7948.path1

* data from the website of https://wheat.pw.usda.gov/GG3/graingenes_downloads/oat-ot3098-pepsico

Supplementary Table 2. Top 50 up/down-regulated DEGs during the oat bracts development.

Top 50 up-regulated DEGs at flowing stage(TCf vs QHf)

Gene ID	L3a.1	L3a.2	L3a.3	L4a.1	L4a.2	L4a.3	L3a_L4a_fc	L3a_L4a_fc2	L3a_L4a_pvalue	L3a_L4a_padj	KEGG	Annotation	Challenger Gene ID	oat-ot3098-pepsico*
avena_sativa_T31230	0	0	0	205	156	122	0.000103509	-13.23795681	5.7E-15	5.99E-12	--	predicted protein [Hordeum vulgare subsp. vulgare]	avena_sativa_T31230	Pepsico1_Contig4748.path1
avena_sativa_T31223	0	0	0	54	65	44	0.000306654	-11.67109873	1.14E-10	4.73E-08	K00422|5.03782e-155|sbi:SORBI_06g025580|K00422 polyphenol oxidase [EC:1.10.3.1] | (RefSeq) SORBIDRAFT_06g025580	Sb06g025580; hypothetical protein polyphenol oxidase [Triticum aestivum]	avena_sativa_T31223	Pepsico2_Contig12951.path1
avena_sativa_T7862	0	0	0	36	41	40	0.000427168	-11.19290922	1.42E-09	0.000000431	NA		avena_sativa_T7862	TRINITY_DN22727_c0_g1_i3.path1
avena_sativa_T1353	0	0	0	30	14	33	0.000648929	-10.58965115	0.000000225	0.0000344	K08245|0|bdi:100839510|K08245 phytepsin [EC:3.4.23.40] | (RefSeq) aspartic proteinase	Aspartic proteinase [Triticum urartu]	avena_sativa_T1353	Pepsico1_Contig21373.path1
avena_sativa_T63067	0	0	0	34	32	11	0.000648929	-10.58965115	0.000000567	0.0000761	K10405|0|bdi:100834158|K10405 kinesin family member C1 | (RefSeq) kinesin-5-like	PREDICTED: kinesin-5-like [Brachypodium distachyon]	avena_sativa_T63067	TRINITY_DN15321_c0_g1_i6.path2
avena_sativa_T10620	0	0	0	24	30	16	0.000713776	-10.45224124	0.000000282	0.0000416	--	Os06g0124900 [Oryza sativa Japonica Group]	avena_sativa_T10620	TRINITY_DN5740_c0_g1_i1.path1
avena_sativa_T19793	0	0	0	13	14	42	0.000724113	-10.4314976	0.00000193	0.000213186	K17991|1.81208e-130|bdi:100840663|K17991 peroxygenase [EC:1.11.2.3] | (RefSeq) probable peroxygenase 5	PREDICTED: probable peroxygenase 5 [Brachypodium distachyon]	avena_sativa_T19793	Pepsico1_Contig112.path1
avena_sativa_T56235	0	0	0	33	13	11	0.000876424	-10.15608308	0.00000545	0.000482924	K00430|1.82905e-161|bdi:100846597|K00430 peroxidase [EC:1.11.1.7] | (RefSeq) peroxidase 72-like	Peroxidase 72 [Triticum urartu]	avena_sativa_T56235	Pepsico1_Contig2963.path2
avena_sativa_T64732	0	0	0	20	18	14	0.000960615	-10.02375435	0.00000189	0.000209802	NA		avena_sativa_T64732	Pepsico1_Contig26711.path1
avena_sativa_T39245	0	0	0	16	14	16	0.001085776	-9.847057346	0.00000404	0.00039386	K20843|1.35023e-38|sbi:SORBI_01g004630|K20843 hydroxyproline O-galactosyltransferase 2/3/4/5/6 [EC:2.4.1.-] | (RefSeq) SORBIDRAFT_01g004630	Sb01g004630; hypothetical protein hypothetical protein SORBIDRAFT_01g004630 [Sorghum bicolor]	avena_sativa_T39245	Pepsico2_Contig20866.path1
avena_sativa_T58097	0	0	0	13	15	17	0.001109878	-9.815383296	0.00000506	0.000460993	K00864|0|bdi:100831505|K00864 glycerol kinase [EC:2.7.1.30] | (RefSeq) glycerol kinase	glycerol kinase [Triticum aestivum]	avena_sativa_T58097	Pepsico1_Contig30909.path1
avena_sativa_T9240	0	0	0	1	44	0	0.001109878	-9.815383296	0.010893019	0.150098408	--	PREDICTED: defensin Tm-AMP-D1.2-like [Brachypodium distachyon]	avena_sativa_T9240	TRINITY_DN6365_c0_g1_i3.path1
avena_sativa_T10796	0	0	0	22	2	20	0.001135074	-9.782998209	0.000206456	0.008841484	--	PREDICTED: cytochrome b5-like [Oryza brachyantha]	avena_sativa_T10796	Pepsico2_Contig20097.path1
avena_sativa_T66968	0	0	0	1	42	0	0.00116144	-9.749869427	0.011920158	0.157798504	K01193|0|obr:102701886|K01193 beta-fructofuranosidase [EC:3.2.1.26] | (RefSeq) sucrose:sucrose 1-fructosyltransferase-like	fructosyltransferase [Phleum pratense]	avena_sativa_T66968	Pepsico1_Contig26692.path1
avena_sativa_T23950	0	0	0	11	18	12	0.001218027	-9.681238412	0.0000139	0.001077116	K04078|6.05959e-144|bdi:100821312|K04078 chaperonin GroES | (RefSeq) 20 kDa chaperonin	chloroplastic-like PREDICTED: 20 kDa chaperonin	avena_sativa_T23950	Pepsico1_Contig6962.path1
avena_sativa_T48021	0	0	0	14	14	12	0.001248439	-9.645658432	0.0000121	0.000966753	NA		avena_sativa_T48021	Pepsico2_Contig3717.path1
avena_sativa_T26571	0	0	0	22	11	6	0.00128041	-9.609178738	0.0000704	0.003774628	--	Transmembrane 9 superfamily member 4 [Aegilops tauschii]	avena_sativa_T26571	TRINITY_DN206_c0_g3_i2.path3
avena_sativa_T5680	0	0	0	13	11	14	0.00131406	-9.571752644	0.0000179	NA	K03248|2.31432e-133|bdi:100845086|K03248 translation initiation factor 3 subunit G | (RefSeq) eukaryotic translation initiation factor 3 subunit G	PREDICTED: eukaryotic translation initiation factor 3 subunit G [Brachypodium distachyon]	avena_sativa_T5680	Pepsico2_Contig17638.path1
avena_sativa_T18758	0	0	0	37	0	0	0.001349528	-9.533329732	0.046958822	NA	K14753|9.54459e-178|bdi:100844071|K14753 guanine nucleotide-binding protein subunit beta-2-like 1 protein | (RefSeq) guanine nucleotide-binding protein subunit beta-like protein A	unnamed protein product [Triticum aestivum]	avena_sativa_T18758	Pepsico2_Contig20564.path1
avena_sativa_T43143	0	0	0	12	15	10	0.001349528	-9.533329732	0.0000255	NA	K08770|4.5749e-48|dosa:Os06t0673500-01|K08770 ubiquitin C | (RAP-DB) Os06g0673500; Similar to polyubiquitin containing 7 ubiquitin monomers.	ubiquitin / ribosomal protein S27a - potato (fragment)	avena_sativa_T43143	Pepsico2_Contig10810.path1
avena_sativa_T10608	0	0	0	17	1	16	0.001468429	-9.411510988	0.001135344	NA	NA		avena_sativa_T10608	NA
avena_sativa_T17510	0	0	0	27	0	6	0.001512859	-9.368506462	0.00977635	NA	NA		avena_sativa_T17510	NA
avena_sativa_T33770	0	0	0	16	8	9	0.001512859	-9.368506462	0.0000841	NA	K00012|0|bdi:100840467|K00012 UDPglucose 6-dehydrogenase [EC:1.1.1.22] | (RefSeq) UDP-glucose 6-dehydrogenase 4-like	PREDICTED: UDP-glucose 6-dehydrogenase 4-like [Brachypodium distachyon]	avena_sativa_T33770	Pepsico2_Contig12020.path1

avena_sativa_T58682	0	0	0	12	12	9	0.001512859	-9.368506462	0.0000535	NA	K00033|0|bdi:100836182|K00033 6-phosphogluconate dehydrogenase [EC:1.1.1.44 1.1.1.343] | (RefSeq) 6-phosphogluconate dehydrogenase	decarboxylating 1 PREDICTED: 6-phosphogluconate dehydrogenase	avena_sativa_T58682	Pepsico2_Contig14972.path1
avena_sativa_T4864	0	0	0	15	12	5	0.001560062	-9.324180547	0.000163891	NA	--	hypothetical protein TRIUR3_25909 [Triticum urartu]	avena_sativa_T4864	Pepsico1_Contig3766.path1
avena_sativa_T55391	0	0	0	14	14	4	0.001560062	-9.324180547	0.000231253	NA	--	predicted protein [Hordeum vulgare subsp. vulgare]	avena_sativa_T55391	Pepsico2_Contig7679.path2
avena_sativa_T18833	0	0	0	9	9	13	0.001610306	-9.278449458	0.0000861	NA	NA		avena_sativa_T18833	TRINITY_DN8242_c0_g4_i1.path1
avena_sativa_T23198	0	0	0	9	9	11	0.00172117	-9.182394353	0.000120526	NA	K13343|3.72796e-16|bdi:100825820|K13343 peroxin-14 | (RefSeq) peroxisomal membrane protein PEX14	Peroxisomal membrane protein PEX14 [Triticum urartu]	avena_sativa_T23198	TRINITY_DN43498_c0_g1_i1.path1
avena_sativa_T54601	0	0	0	1	27	0	0.001782531	-9.131856961	0.026871467	NA	--	meiosis 5 [Triticum aestivum]	avena_sativa_T54601	Pepsico2_Contig10332.path1
avena_sativa_T11777	0	0	0	10	10	6	0.001919386	-9.025139562	0.000311839	NA	NA		avena_sativa_T11777	Pepsico1_Contig33280.path1
avena_sativa_T5077	0	0	0	3	23	0	0.001919386	-9.025139562	0.021021366	NA	--	PREDICTED: expansin-B4 [Brachypodium distachyon]	avena_sativa_T5077	Pepsico1_Contig3199.path1
avena_sativa_T52192	0	0	0	10	8	8	0.001919386	-9.025139562	0.000253752	NA	--	predicted protein [Hordeum vulgare subsp. vulgare]	avena_sativa_T52192	Pepsico2_Contig11530.path1
avena_sativa_T55671	0	0	0	7	9	10	0.001919386	-9.025139562	0.00026321	NA	K03255|0|bdi:100830075|K03255 protein TIF31 | (RefSeq) protein TSS	hypothetical protein F775_09910 [Aegilops tauschii]	avena_sativa_T55671	Pepsico2_Contig16552.path1
avena_sativa_T9939	0	0	0	7	9	10	0.001919386	-9.025139562	0.00026321	NA	NA		avena_sativa_T9939	TRINITY_DN6501_c0_g1_i1.path1
avena_sativa_T52928	0	0	0	5	19	1	0.001996008	-8.968666793	0.004600786	NA	K11251|1.5355e-49|zma:103639303|K11251 histone H2A | (RefSeq) his2a1	GRMZM2G305046; histone2A1 Histone H2A [Triticum urartu]	avena_sativa_T52928	TRINITY_DN2145_c0_g1_i1.path2
avena_sativa_T7389	0	0	0	1	1	23	0.001996008	-8.968666793	0.012072582	NA	--	hypothetical protein TRIUR3_03033 [Triticum urartu]	avena_sativa_T7389	Pepsico2_Contig8228.path1
avena_sativa_T42711	0	0	0	8	8	8	0.002079002	-8.909893084	0.000398282	NA	--	PREDICTED: LOW QUALITY PROTEIN: formin-like protein 5 [Brachypodium distachyon]	avena_sativa_T42711	Pepsico2_Contig16560.path1
avena_sativa_T12366	0	0	0	4	19	0	0.002169197	-8.84862294	0.02415467	NA	K11254|7.88647e-50|zma:100282268|K11254 histone H4 | (RefSeq) pco082380(357)	GRMZM2G056350 GRMZM2G332838; uncharacterized LOC100282268	avena_sativa_T12366	TRINITY_DN21711_c0_g1_i1.path2
avena_sativa_T57819	0	0	0	1	9	13	0.002169197	-8.84862294	0.004177563	NA	--	Cell wall-associated hydrolase partial [Medicago truncatula]	avena_sativa_T57819	Pepsico2_Contig17422.path5
avena_sativa_T10413	0	0	0	3	19	0	0.002267574	-8.784634846	0.02911569	NA	K11254|2.09546e-49|zma:100282268|K11254 histone H4 | (RefSeq) pco082380(357)	GRMZM2G056350 GRMZM2G332838; uncharacterized LOC100282268	avena_sativa_T10413	TRINITY_DN27605_c0_g1_i2.path1
avena_sativa_T46781	0	0	0	5	5	12	0.002267574	-8.784634846	0.001274714	NA	--	OSJNBa0087O24.13 [Oryza sativa Japonica Group]	avena_sativa_T46781	Pepsico2_Contig20979.path1
avena_sativa_T5327	0	0	0	1	21	0	0.002267574	-8.784634846	0.041301408	NA	--	14 kDa proline-rich protein DC2.15 [Triticum urartu]	avena_sativa_T5327	Pepsico1_Contig8167.path1
avena_sativa_T54420	0	0	0	7	8	7	0.002267574	-8.784634846	0.000691802	NA	NA		avena_sativa_T54420	TRINITY_DN28365_c0_g1_i3.path1
avena_sativa_T10205	0	0	0	1	9	11	0.002375297	-8.717676423	0.005399158	NA	--	PREDICTED: uncharacterized protein LOC100840728 [Brachypodium distachyon]	avena_sativa_T10205	TRINITY_DN74454_c0_g1_i1.path1
avena_sativa_T11717	0	0	0	7	8	6	0.002375297	-8.717676423	0.00094736	NA	K14565|1.92044e-07|dosa:Os11t0580500-00|K14565 nucleolar protein 58 | (RAP-DB) Os11g0580500; NOP5	N-terminal domain containing protein. hypothetical protein TRIUR3_00178 [Triticum urartu]	avena_sativa_T11717	TRINITY_DN26246_c0_g2_i2.path1
avena_sativa_T66928	0	0	0	8	8	5	0.002375297	-8.717676423	0.001066098	NA	--	Protein IQ-DOMAIN 1 [Triticum urartu]	avena_sativa_T66928	Pepsico2_Contig20251.path2
avena_sativa_T41041	0	0	0	8	3	9	0.002493766	-8.647458426	0.002203722	NA	--	predicted protein [Hordeum vulgare subsp. vulgare]	avena_sativa_T41041	Pepsico1_Contig28400.path1
avena_sativa_T5024	0	0	0	1	19	0	0.002493766	-8.647458426	0.04865293	NA	K08235|5.25355e-172|obr:102717793|K08235 xyloglucan:xyloglucosyl transferase [EC:2.4.1.207] | (RefSeq) xyloglucan endotransglucosylase/hydrolase protein 9	predicted protein [Hordeum vulgare subsp. vulgare]	avena_sativa_T5024	Pepsico1_Contig6608.path1
avena_sativa_T54792	0	0	0	7	6	7	0.002493766	-8.647458426	0.001209293	NA	NA		avena_sativa_T54792	TRINITY_DN20932_c0_g1_i3.path1
avena_sativa_T18429	0	0	0	5	5	9	0.002624672	-8.573647187	0.002037189	NA	K12191|4.1091e-103|bdi:100840560|K12191 charged multivesicular body protein 2A | (RefSeq) vacuolar protein sorting-associated protein 2 homolog 1-like	Vacuolar protein sorting-associated protein 2-like protein 1 [Triticum urartu]	avena_sativa_T18429	Pepsico1_Contig3545.path1

* data from the website of https://wheat.pw.usda.gov/GG3/graingenes downloads/oat-ot3098-pepsico

Supplementary Table 2. Top 50 up/down-regulated DEGs during the oat bracts development.

Top 50 up-regulated DEGs at milk stage(TCm vs QHm)

Gene ID	L3a.1	L3a.2	L3a.3	L4a.1	L4a.2	L4a.3	L3a_L4a_fc	L3a_L4a_fc2	L3a_L4a_pvalue	L3a_L4a_padj	KEGG	Annotation	Challenger Gene ID	oat-ot3098-pepsico*
avena_sativa_T53549	0	0	0	170	100	267	0.0000931	-13.3908407	1.07E-14	1.85E-11	NA		avena_sativa_T53549	Pepsico1_Contig8164.path1
avena_sativa_T9476	0	0	0	92	82	136	0.000161264	-12.59828517	3.54E-13	4.7E-10	--	RecName: Full=Thioredoxin H-type; Short=Trx-H; AltNam	avena_sativa_T9476	Pepsico1_Contig2129.path1
avena_sativa_T21726	0	0	0	21	87	0	0.000462749	-11.07748336	0.049133368	0.989955604	NA		avena_sativa_T21726	Pepsico1_Contig25537.path1
avena_sativa_T1353	0	0	0	29	29	42	0.00049975	-10.96650545	1.06E-08	0.00000617	K08245|0|bdi:100839510|K08245 phytepsin [EC:3.4.23.40] | (RefSeq) aspartic proteinase	Aspartic proteinase [Triticum urartu]	avena_sativa_T1353	Pepsico1_Contig21373.path1
avena_sativa_T20559	0	0	0	19	18	52	0.000561482	-10.7984718	0.000000201	0.0000825	--	Os12g0115000 [Oryza sativa Japonica Group]	avena_sativa_T20559	Pepsico2_Contig14652.path1
avena_sativa_T22652	0	0	0	31	27	30	0.000567859	-10.78217919	2.42E-08	0.0000128	K15171|1.21455e-71|zma:100284208|K15171 transcription elongation factor SPT4 | (RefSeq) GRMZM2G086805; transcription elongation factor SPT4	predicted protein [Hordeum vulgare subsp. vulgare]	avena_sativa_T22652	Pepsico2_Contig221.path1
avena_sativa_T60750	0	0	0	20	20	41	0.000616903	-10.66266838	0.000000152	0.000067	K04121|0|sita:101772220|K04121 ent-kaurene synthase [EC:4.2.3.19] | (RefSeq) ent-kaur-16-ene synthase	chloroplastic-like predicted protein [Hordeum vulgare subs	avena_sativa_T60750	NA
avena_sativa_T10620	0	0	0	27	17	31	0.000666223	-10.55170826	0.000000165	0.0000709	--	Os06g0124900 [Oryza sativa Japonica Group]	avena_sativa_T10620	TRINITY_DN5740_c0_g1_i1.path1
avena_sativa_T7076	0	0	0	27	23	25	0.000666223	-10.55170826	9.95E-08	0.0000454	--	predicted protein [Hordeum vulgare subsp. vulgare]	avena_sativa_T7076	TRINITY_DN4954_c0_g3_i10.path1
avena_sativa_T10796	0	0	0	21	20	26	0.000745712	-10.38909352	0.000000282	0.000112248	--	PREDICTED: cytochrome b5-like [Oryza brachyantha]	avena_sativa_T10796	Pepsico2_Contig20097.path1
avena_sativa_T66221	0	0	0	19	28	16	0.000793021	-10.30035256	0.000000749	0.000254393	--	hypothetical protein F775_09400 [Aegilops tauschii]	avena_sativa_T66221	TRINITY_DN5239_c0_g3_i1.path2
avena_sativa_T22886	0	0	0	55	1	2	0.000861326	-10.18115226	0.000849242	0.087258793	--	globulin-3A [Triticum aestivum]	avena_sativa_T22886	Pepsico2_Contig19099.path1
avena_sativa_T22843	0	0	0	26	10	19	0.000908265	-10.10459875	0.00000358	0.000948533	NA		avena_sativa_T22843	TRINITY_DN58605_c0_g1_i1.path2
avena_sativa_T25493	0	0	0	13	13	24	0.000999001	-9.967226259	0.00000516	0.00130645	--	predicted protein [Hordeum vulgare subsp. vulgare]	avena_sativa_T25493	TRINITY_DN11581_c0_g1_i1.path5
avena_sativa_T25920	0	0	0	13	6	29	0.001040583	-9.908392621	0.0000301	0.005813811	K00789|0|bdi:100844978|K00789 S-adenosylmethionine synthetase [EC:2.5.1.6] | (RefSeq) S-adenosylmethionine synthase 3-like	RecName: Full=S-adenosylmethionine synthase 3; Short=A	avena_sativa_T25920	Pepsico2_Contig11347.path1
avena_sativa_T40826	0	0	0	13	27	8	0.001040583	-9.908392621	0.0000178	0.003853824	--	predicted protein [Hordeum vulgare subsp. vulgare]	avena_sativa_T40826	Pepsico2_Contig6159.path1
avena_sativa_T49117	0	0	0	11	7	26	0.001135074	-9.782998209	0.0000369	0.006986927	K04120|9.30463e-66|zma:103641248|K04120 ent-copalyl diphosphate synthase [EC:5.5.1.13] | (RefSeq) GRMZM2G068808; syn-copalyl diphosphate synthase-like	PREDICTED: syn-copalyl diphosphate synthase-like isofor	avena_sativa_T49117	TRINITY_DN59316_c0_g1_i1.path5
avena_sativa_T21832	0	0	0	10	16	17	0.00116144	-9.749869427	0.0000131	0.00294866	--	predicted protein [Hordeum vulgare subsp. vulgare]	avena_sativa_T21832	TRINITY_DN41013_c0_g1_i1.path1
avena_sativa_T58508	0	0	0	3	38	1	0.001189061	-9.71596199	0.001388467	0.123672388	--	predicted protein [Hordeum vulgare subsp. vulgare]	avena_sativa_T58508	Pepsico1_Contig880.path1
avena_sativa_T5299	0	0	0	39	0	0	0.00128041	-9.609178738	0.028730365	0.7937235	--	meiosis 5 [Triticum aestivum]	avena_sativa_T5299	Pepsico1_Contig4128.path1
avena_sativa_T50144	0	0	0	12	12	14	0.00131406	-9.571752644	0.0000245	0.005053906	--	Os08g0233900 [Oryza sativa Japonica Group]	avena_sativa_T50144	Pepsico1_Contig34123.path3
avena_sativa_T36983	0	0	0	5	31	0	0.001386963	-9.493855449	0.005701096	0.313729263	--	hypothetical protein F775_32644 [Aegilops tauschii]	avena_sativa_T36983	TRINITY_DN13294_c0_g1_i1.path1
avena_sativa_T9894	0	0	0	35	0	0	0.001426534	-9.453270634	0.035251551	0.864827694	K11251|8.38828e-48|zma:100501984|K11251 histone H2A | (RefSeq) GRMZM2G003306; uncharacterized LOC100501984	predicted protein [Hordeum vulgare subsp. vulgare]	avena_sativa_T9894	TRINITY_DN26227_c0_g1_i2.path2
avena_sativa_T63606	0	0	0	23	10	0	0.001512859	-9.368506462	0.005550356	0.309099342	--	hypothetical protein TRIUR3_06539 [Triticum urartu]	avena_sativa_T63606	TRINITY_DN1753_c0_g1_i1.path1
avena_sativa_T64812	0	0	0	12	12	8	0.001560062	-9.324180547	0.0000977	0.015997505	NA		avena_sativa_T64812	Pepsico1_Contig26990.path1
avena_sativa_T10359	0	0	0	22	8	0	0.001663894	-9.231221181	0.007808903	0.376843834	--	PREDICTED: uncharacterized protein LOC100837178 [Bra	avena_sativa_T10359	NA
avena_sativa_T33754	0	0	0	3	9	16	0.001782531	-9.131856961	0.000730541	0.079460557	NA		avena_sativa_T33754	TRINITY_DN37850_c0_g4_i3.path1
avena_sativa_T23198	0	0	0	9	8	10	0.001848429	-9.079484784	0.000265971	0.036126683	K13343|3.72796e-16|bdi:100825820|K13343 peroxin-14 | (RefSeq) peroxisomal membrane protein PEX14	Peroxisomal membrane protein PEX14 [Triticum urartu]	avena_sativa_T23198	TRINITY_DN43498_c0_g1_i1.path1
avena_sativa_T34732	0	0	0	8	11	8	0.001848429	-9.079484784	0.000285032	0.0376148	--	predicted protein [Hordeum vulgare subsp. vulgare]	avena_sativa_T34732	Pepsico2_Contig5690.path1
avena_sativa_T40625	0	0	0	12	15	0	0.001848429	-9.079484784	0.009020607	0.41244466	K15015|0|bdi:100822506|K15015 solute carrier family 32 (vesicular inhibitory amino acid transporter) | (RefSeq) vacuolar amino acid transporter 1-like	hypothetical protein F775_07363 [Aegilops tauschii]	avena_sativa_T40625	Pepsico2_Contig3776.path1
avena_sativa_T54366	0	0	0	9	9	9	0.001848429	-9.079484784	0.0002585	0.035283959	NA		avena_sativa_T54366	TRINITY_DN81998_c0_g1_i1.path1
avena_sativa_T5569	0	0	0	8	6	13	0.001848429	-9.079484784	0.000375382	0.047728399	--	predicted protein [Hordeum vulgare subsp. vulgare]	avena_sativa_T5569	TRINITY_DN72984_c0_g1_i1.path1
avena_sativa_T14984	0	0	0	21	3	0	0.002079002	-8.909893084	0.019621235	0.633812075	--	2OG-Fe(II) oxygenase [Lolium perenne]	avena_sativa_T14984	Pepsico1_Contig6738.path1
avena_sativa_T44382	0	0	0	12	11	1	0.002079002	-8.909893084	0.003207752	0.219999643	NA		avena_sativa_T44382	TRINITY_DN33640_c0_g3_i1.path1
avena_sativa_T10243	0	0	0	14	9	0	0.002169197	-8.84862294	0.01491455	0.548812105	--	PREDICTED: uncharacterized protein LOC100828693 [Bra	avena_sativa_T10243	TRINITY_DN12_c0_g2_i1.path1
avena_sativa_T24078	0	0	0	8	10	5	0.002169197	-8.84862294	0.000898481	0.090610632	K17732|2.61937e-63|bdi:100844576|K17732 mitochondrial-processing peptidase subunit beta [EC:3.4.24.64] | (RefSeq) probable mitochondrial-processing peptidase subunit beta	unnamed protein product [Triticum aestivum]	avena_sativa_T24078	Pepsico2_Contig511.path1
avena_sativa_T24623	0	0	0	9	14	0	0.002169197	-8.84862294	0.014611945	0.54394331	K15078|3.86117e-136|bdi:100832723|K15078 structure-specific endonuclease subunit SLX1 [EC:3.6.1.-] | (RefSeq) uncharacterized LOC100832723	predicted protein [Hordeum vulgare subsp. vulgare]	avena_sativa_T24623	TRINITY_DN46573_c0_g1_i1.path1
avena_sativa_T35603	0	0	0	7	16	0	0.002169197	-8.84862294	0.015413575	0.558115738	--	PREDICTED: uncharacterized protein LOC101754494 [Set	avena_sativa_T35603	NA

avena_sativa_T30290	0	0	0	6	5	10	0.002375297	-8.717676423	0.001533486	0.133855506	--	predicted protein [Hordeum vulgare subsp. vulgare]	avena_sativa_T30290	Pepsico1_Contig19974.path5
avena_sativa_T38808	0	0	0	3	11	7	0.002375297	-8.717676423	0.002233892	0.175218962	NA		avena_sativa_T38808	NA
avena_sativa_T33707	0	0	0	16	4	0	0.002493766	-8.647458426	0.026572883	0.755404234	--	2OG-Fe(II) oxygenase [Lolium perenne]	avena_sativa_T33707	Pepsico1_Contig6738.path1
avena_sativa_T56086	0	0	0	5	7	8	0.002493766	-8.647458426	0.001746926	0.146958156	--	putative gag-pol polyprotein [Oryza sativa Japonica Group]	avena_sativa_T56086	TRINITY_DN6205_c1_g1_i1.path1
avena_sativa_T16343	0	0	0	5	6	8	0.002624672	-8.573647187	0.002303382	0.179155504	NA		avena_sativa_T16343	NA
avena_sativa_T13922	0	0	0	4	14	0	0.002770083	-8.495855027	0.031793289	0.834418274	--	hypothetical protein F775_31526 [Aegilops tauschii]	avena_sativa_T13922	NA
avena_sativa_T31896	0	0	0	10	7	0	0.002932551	-8.413627929	0.033138803	0.845904686	--	unnamed protein product [Triticum aestivum]	avena_sativa_T31896	Pepsico1_Contig26746.path2
avena_sativa_T39833	0	0	0	6	5	6	0.002932551	-8.413627929	0.003813191	0.245215502	K08232|3.73583e-142|bdi:100825974|K08232 monodehydroascorbate reductase (NADH) [EC:1.6.5.4] | (RefSeq) monodehydroascorbate reductase	PREDICTED: monodehydroascorbate reductase [Brachypod	avena_sativa_T39833	Pepsico2_Contig331.path1
avena_sativa_T47283	0	0	0	7	10	0	0.002932551	-8.413627929	0.032611374	0.843717593	K00844|0|bdi:100832143|K00844 hexokinase [EC:2.7.1.1] | (RefSeq) hexokinase-7	RecName: Full=Hexokinase-7; AltName: Full=Hexokinase-	avena_sativa_T47283	Pepsico2_Contig5650.path1
avena_sativa_T54759	0	0	0	3	13	1	0.002932551	-8.413627929	0.013651432	0.523647775	NA		avena_sativa_T54759	TRINITY_DN31603_c0_g2_i2.path1
avena_sativa_T7265	0	0	0	4	13	0	0.002932551	-8.413627929	0.036043381	0.871204814	--	hypothetical protein TRIUR3_17102 [Triticum urartu]	avena_sativa_T7265	NA
avena_sativa_T11790	0	0	0	4	10	2	0.003115265	-8.326429487	0.009495363	NA	--	hypothetical protein TRIUR3_01630 [Triticum urartu]	avena_sativa_T11790	Pepsico2_Contig15013.path1

* data from the website of https://wheat.pw.usda.gov/GG3/graingenes_downloads/oat-ot3098-pepsico

Supplementary Table 2. Top 50 up/down-regulated DEGs during the oat bracts development.

Top 50 down-regulated DEGs at heading stage(TCh vs QHh)

Gene ID	L3a.1	L3a.2	L3a.3	L4a.1	L4a.2	L4a.3	L3a_L4a_fc	L3a_L4a_fc2	L3a_L4a_pvalue	L3a_L4a_padj	KEGG	Annotation	Challenger Gene ID	oat-ot3098-pepsico*
avena_sativa_T34359	0	383	621	0	0	0	20081	14.29354349	0.005488073	0.094708606	K00695|0|bdi:100840503|K00695 sucrose synthase [EC:2.4.1.13] | (RefSeq) sucrose synthase 1	predicted protein [Hordeum vulgare subsp. vulgare]	avena_sativa_T34359	Pepsico2_Contig21425.path3
avena_sativa_T6406	0	255	314	0	0	0	11381	13.47433971	0.010255941	0.142566747	K08910|7.97399e-153|bdi:100830344|K08910 light-harvesting complex I chlorophyll a/b binding protein 4 | (RefSeq) chlorophyll a-b binding protein 4	Lhca4,chloroplastic PREDICTED: chlorophyll a-b binding protein 4	avena_sativa_T6406	Pepsico1_Contig8179.path1
avena_sativa_T7746	133	195	181	0	0	0	10181	13.31359165	5.68E-16	5.57E-13	NA		avena_sativa_T7746	TRINITY_DN8506_c1_g1_i1.path1
avena_sativa_T6255	147	128	171	0	0	0	8921	13.12298972	1.96E-15	1.52E-12	NA		avena_sativa_T6255	Pepsico2_Contig2416.path1
avena_sativa_T5397	152	102	109	0	0	0	7261	12.82595254	3.67E-14	2.21E-11	NA		avena_sativa_T5397	Pepsico1_Contig849.path2
avena_sativa_T7421	283	16	0	0	0	0	5981	12.546171	0.018899992	0.211589853	--	predicted protein [Hordeum vulgare subsp. vulgare]	avena_sativa_T7421	NA
avena_sativa_T16471	99	86	109	0	0	0	5881	12.52184578	1.52E-13	8.32E-11	--	hypothetical protein F775_43951 [Aegilops tauschii]	avena_sativa_T16471	TRINITY_DN526_c0_g1_i2.path1
avena_sativa_T41595	281	11	0	0	0	0	5841	12.51199967	0.019333529	0.213918728	--	unnamed protein product [Triticum aestivum]	avena_sativa_T41595	NA
avena_sativa_T60490	0	150	130	0	0	0	5601	12.45146871	0.021167932	0.226556385	K15404|5.97059e-103|bdi:100833295|K15404 aldehyde decarbonylase [EC:4.1.99.5] | (RefSeq) protein ECERIFERUM 1-like	PREDICTED: protein ECERIFERUM 1-like [Brachypodium distachyon]	avena_sativa_T60490	Pepsico2_Contig6620.path1
avena_sativa_T20709	0	145	130	0	0	0	5501	12.42547819	0.021543958	0.228613503	K15404|0|bdi:100833807|K15404 aldehyde decarbonylase [EC:4.1.99.5] | (RefSeq) protein ECERIFERUM 1-like	PREDICTED: protein ECERIFERUM 1-like [Brachypodium distachyon]	avena_sativa_T20709	Pepsico2_Contig6620.path1
avena_sativa_T2622	0	143	128	0	0	0	5421	12.40434329	0.021854014	0.231245959	K20660|0|bdi:100837734|K20660 cytochrome P450 family 709 | (RefSeq) cytochrome P450 734A1-like	PREDICTED: cytochrome P450 734A1-like [Brachypodium distachyon]	avena_sativa_T2622	NA
avena_sativa_T6842	66	80	108	0	0	0	5081	12.31089675	1.36E-12	6.7E-10	NA		avena_sativa_T6842	Pepsico2_Contig18164.path1
avena_sativa_T13114	77	76	96	0	0	0	4981	12.2822197	8.71E-13	4.51E-10	NA		avena_sativa_T13114	TRINITY_DN22727_c0_g1_i3.path1
avena_sativa_T7759	222	13	0	0	0	0	4701	12.19875197	0.023920613	0.246540901	--	fasciclin-like arabinogalactan precursor [Triticum aestivum]	avena_sativa_T7759	NA
avena_sativa_T44728	210	10	0	0	0	0	4401	12.10361566	0.025470311	0.256698349	--	Pollen-specific protein SF3 [Aegilops tauschii]	avena_sativa_T44728	NA
avena_sativa_T23359	208	11	0	0	0	0	4381	12.0970445	0.025587266	0.257302037	--	predicted protein [Hordeum vulgare subsp. vulgare]	avena_sativa_T23359	NA
avena_sativa_T3632	0	145	64	0	0	0	4181	12.02963233	0.02803301	0.271880288	--	Curcuminoid synthase [Triticum urartu]	avena_sativa_T3632	Pepsico2_Contig19170.path1
avena_sativa_T49334	53	60	68	0	0	0	3621	11.82217246	2.18E-11	9.48E-09	--	hypothetical protein F775_29669 [Aegilops tauschii]	avena_sativa_T49334	Pepsico2_Contig13183.path1
avena_sativa_T20589	0	89	91	0	0	0	3601	11.81418188	0.032228224	0.296711853	--	predicted protein [Hordeum vulgare subsp. vulgare]	avena_sativa_T20589	Pepsico2_Contig11082.path1
avena_sativa_T3556	171	9	0	0	0	0	3601	11.81418188	0.030781887	0.288297395	--	pollen allergen Ave s 5 (isoallergen B) [Avena sativa]	avena_sativa_T3556	Pepsico1_Contig4165.path1
avena_sativa_T24022	171	7	0	0	0	0	3561	11.79806672	0.03108772	0.290039617	K01728|0|bdi:100829073|K01728 pectate lyase [EC:4.2.2.2] | (RefSeq) pectate lyase-like	predicted protein [Hordeum vulgare subsp. vulgare]	avena_sativa_T24022	TRINITY_DN85129_c0_g1_i1.path2
avena_sativa_T36543	166	7	0	0	0	0	3461	11.75697323	0.031922019	0.295096757	--	PREDICTED: GPI-anchored protein LORELEI-like [Brachypodium distachyon]	avena_sativa_T36543	TRINITY_DN89660_c0_g1_i2.path1
avena_sativa_T26549	60	53	51	0	0	0	3281	11.67991988	6.04E-11	2.28E-08	--	helicase-like protein [Oryza sativa Japonica Group]	avena_sativa_T26549	Pepsico1_Contig27196.path1
avena_sativa_T47818	155	6	0	0	0	0	3221	11.65329295	0.034103301	0.30645656	--	pollen allergen Ave s 5 (isoallergen B) [Avena sativa]	avena_sativa_T47818	Pepsico1_Contig4165.path1
avena_sativa_T4793	150	8	0	0	0	0	3161	11.62616532	0.03471758	0.310069023	--	pollen allergen Ave s 5 (isoallergen B) [Avena sativa]	avena_sativa_T4793	Pepsico1_Contig7995.path1
avena_sativa_T6402	0	72	86	0	0	0	3161	11.62616532	0.036319075	0.315860374	--	hypothetical protein F775_43951 [Aegilops tauschii]	avena_sativa_T6402	TRINITY_DN526_c0_g1_i2.path1
avena_sativa_T5741	56	17	80	0	0	0	3061	11.57978733	1.24E-08	0.00000285	NA		avena_sativa_T5741	Pepsico1_Contig7932.path1
avena_sativa_T7022	0	76	77	0	0	0	3061	11.57978733	0.037393825	0.321762828	--	PREDICTED: TATA-binding protein 2-like isoform X1 [Brachypodium distachyon]	avena_sativa_T7022	TRINITY_DN80635_c0_g1_i1.path1
avena_sativa_T3784	144	8	0	0	0	0	3041	11.5703301	0.035969345	0.314879463	K00430|2.31487e-177|bdi:100834380|K00430 peroxidase [EC:1.11.1.7] | (RefSeq) peroxidase 44-like	Peroxidase 44 [Aegilops tauschii]	avena_sativa_T3784	Pepsico1_Contig6129.path1
avena_sativa_T32107	65	43	43	0	0	0	3021	11.56081047	3.06E-10	9.84E-08	--	hypothetical protein F775_32419 [Aegilops tauschii]	avena_sativa_T32107	Pepsico1_Contig4308.path1
avena_sativa_T7928	0	77	73	0	0	0	3001	11.5512276	0.03806907	0.324850872	NA		avena_sativa_T7928	Pepsico2_Contig15160.path1
avena_sativa_T45153	0	74	67	0	0	0	2821	11.46199095	0.040246281	0.334912915	--	PREDICTED: thioredoxin H-type-like [Brachypodium distachyon]	avena_sativa_T45153	Pepsico1_Contig1731.path1
avena_sativa_T34674	0	60	74	0	0	0	2681	11.3885555	0.042116556	0.342346122	K15397|0|bdi:100824744|K15397 3-ketoacyl-CoA synthase [EC:2.3.1.199] | (RefSeq) 3-ketoacyl-CoA synthase 6-like	3-ketoacyl-CoA synthase 6 [Aegilops tauschii]	avena_sativa_T34674	NA
avena_sativa_T56825	127	6	0	0	0	0	2661	11.3777528	0.040560316	0.335842294	--	predicted protein [Hordeum vulgare subsp. vulgare]	avena_sativa_T56825	NA
avena_sativa_T10441	126	6	0	0	0	0	2641	11.36686859	0.040835454	0.337073182	--	hypothetical protein SORBIDRAFT_03g046870 [Sorghum bicolor]	avena_sativa_T10441	NA
avena_sativa_T36994	57	20	55	0	0	0	2641	11.36686859	9.38E-09	0.00000227	NA		avena_sativa_T36994	TRINITY_DN30558_c0_g1_i2.path1
avena_sativa_T6226	125	5	0	0	0	0	2601	11.34485068	0.041382489	0.338889526	--	predicted protein [Hordeum vulgare subsp. vulgare]	avena_sativa_T6226	Pepsico1_Contig7768.path1
avena_sativa_T44949	119	7	0	0	0	0	2521	11.2997804	0.042581944	0.343881453	--	PREDICTED: uncharacterized protein LOC100834442 [Brachypodium distachyon]	avena_sativa_T44949	NA
avena_sativa_T39457	20	58	47	0	0	0	2501	11.28828934	1.04E-08	0.00000246	K02927|2.9595e-86|bdi:100828943|K02927 large subunit ribosomal protein L40e | (RefSeq) ubiquitin-60S ribosomal protein L40-2	PREDICTED: ubiquitin-60S ribosomal protein L40-2 [Brachypodium distachyon]	avena_sativa_T39457	TRINITY_DN6478_c0_g1_i3.path1
avena_sativa_T10314	0	66	57	0	0	0	2461	11.26502894	0.045428883	0.35766958	--	hypothetical protein TRIUR3_01588 [Triticum urartu]	avena_sativa_T10314	TRINITY_DN20164_c0_g1_i1.path1
avena_sativa_T30455	0	72	51	0	0	0	2461	11.26502894	0.045428883	0.35766958	NA		avena_sativa_T30455	Pepsico1_Contig2554.path1

avena_sativa_T6941	113	6	0	0	0	0	2381	11.21735191	0.044779005	0.355000917	--	PREDICTED: LIM domain-containing protein PLIM2b-like [Brachypodium distachyon]	avena_sativa_T6941	NA
avena_sativa_T11850	109	6	0	0	0	0	2301	11.16804527	0.046148433	0.361722275	--	PREDICTED: pectinesterase-like [Brachypodium distachyon]	avena_sativa_T11850	Pepsico1_Contig21931.path1
avena_sativa_T46451	106	8	0	0	0	0	2281	11.15545073	0.046540194	0.363875657	NA		avena_sativa_T46451	NA
avena_sativa_T54168	25	45	42	0	0	0	2241	11.12992693	5.91E-09	0.00000147	NA		avena_sativa_T54168	TRINITY_DN16761_c0_g1_i1.path1
avena_sativa_T12141	0	58	53	0	0	0	2221	11.11699368	0.049685974	0.374956248	K14457|6.0278e-33|obr:102707405|K14457 2-acylglycerol O-acyltransferase 2 [EC:2.3.1.22] | (RefSeq) diacylglycerol O-acyltransferase 2	unnamed protein product [Oryza sativa Japonica Group]	avena_sativa_T12141	NA
avena_sativa_T9270	101	5	0	0	0	0	2121	11.05052891	0.049535529	0.374424138	--	hypothetical protein OsJ_30442 [Oryza sativa Japonica Group]	avena_sativa_T9270	NA
avena_sativa_T12847	100	5	0	0	0	0	2101	11.03686045	0.049945009	0.375846774	--	pollen allergen [Lolium perenne]	avena_sativa_T12847	Pepsico1_Contig5618.path1
avena_sativa_T2964	30	23	44	0	0	0	1941	10.9225844	2.71E-08	0.00000557	--	hypothetical protein OsI_29466 [Oryza sativa Indica Group]	avena_sativa_T2964	Pepsico1_Contig24192.path1
avena_sativa_T1269	90	4	1	0	0	0	1901	10.89254282	0.000360732	0.013990535	--	unnamed protein product [Triticum aestivum]	avena_sativa_T1269	Pepsico2_Contig17320.path1
														* data from the website of https://wheat.pw.usda.gov/GG3/graingenes_downloads/oat-ot3098-pepsico

Supplementary Table 2. Top 50 up/down-regulated DEGs during the oat bracts development.

Top 50 down-regulated DEGs at flowing stage(TCf vs QHf)

Gene ID	L3a.1	L3a.2	L3a.3	L4a.1	L4a.2	L4a.3	L3a_L4a_fc	L3a_L4a_fc2	L3a_L4a_pvalue	L3a_L4a_padj	KEGG	Annotation	Challenger Gene ID	oat-ot3098-pepsico*
avena_sativa_T5397	347	349	143	0	0	0	16781	14.03454107	1.62E-16	2.22E-13	NA		avena_sativa_T5397	Pepsico1_Contig849.path2
avena_sativa_T6406	2	35	548	0	0	0	11701	13.51434421	0.000602584	0.019890849	K08910|7.97399e-153|bdi:100830344|K08910 light-harvesting complex I chlorophyll a/b binding protein 4 | (RefSeq) chlorophyll a-b binding protein 4	Lhca4,chloroplastic PREDICTED: chlorophyll a-b binding protein 4	avena_sativa_T6406	Pepsico1_Contig8179.path1
avena_sativa_T13114	192	171	121	0	0	0	9681	13.24094036	2.64E-15	3.14E-12	NA		avena_sativa_T13114	TRINITY_DN22727_c0_g1_i3.path1
avena_sativa_T7928	1	15	257	0	0	0	5461	12.41494944	0.0025054	0.055569545	NA		avena_sativa_T7928	Pepsico2_Contig15160.path1
avena_sativa_T6255	110	137	11	0	0	0	5161	12.33343492	5.3E-09	0.00000139	NA		avena_sativa_T6255	Pepsico2_Contig2416.path1
avena_sativa_T54484	73	89	77	0	0	0	4781	12.22309669	1.25E-12	7.06E-10	--	hypothetical protein F775_29174 [Aegilops tauschii]	avena_sativa_T54484	Pepsico2_Contig7287.path1
avena_sativa_T49334	76	69	69	0	0	0	4281	12.06373212	3.36E-12	1.77E-09	--	hypothetical protein F775_29669 [Aegilops tauschii]	avena_sativa_T49334	Pepsico2_Contig13183.path1
avena_sativa_T38298	57	56	53	0	0	0	3321	11.69740201	3.76E-11	1.68E-08	K08770|2.92726e-156|aly:ARALYDRAFT_490270|K08770 ubiquitin C | (RefSeq) hypothetical protein	Multidrug resistance protein ABC transporter family [Medicago truncatula]	avena_sativa_T38298	Pepsico1_Contig201.path1
avena_sativa_T6842	47	41	62	0	0	0	3001	11.5512276	1.71E-10	6.23E-08	NA		avena_sativa_T6842	Pepsico2_Contig18164.path1
avena_sativa_T62702	45	51	47	0	0	0	2861	11.48230378	1.57E-10	5.85E-08	K02927|3.75425e-85|bdi:100828943|K02927 large subunit ribosomal protein L40e | (RefSeq) ubiquitin-60S ribosomal protein L40-2	PREDICTED: ubiquitin-60S ribosomal protein L40-2 [Brachypodium distachyon]	avena_sativa_T62702	TRINITY_DN6478_c0_g1_i3.path1
avena_sativa_T26549	1	60	58	0	0	0	2381	11.21735191	0.0000126	0.000989013	--	helicase-like protein [Oryza sativa Japonica Group]	avena_sativa_T26549	Pepsico1_Contig27196.path1
avena_sativa_T54168	35	32	46	0	0	0	2261	11.14274528	0.000000002	0.000000585	NA		avena_sativa_T54168	TRINITY_DN16761_c0_g1_i1.path1
avena_sativa_T30455	0	6	105	0	0	0	2221	11.11699368	0.049605765	0.354808795	NA		avena_sativa_T30455	Pepsico1_Contig2554.path1
avena_sativa_T52545	51	41	19	0	0	0	2221	11.11699368	1.37E-08	0.00000322	K03177|0|osa:4337577|K03177 tRNA pseudouridine55 synthase [EC:5.4.99.25] | (RefSeq) uncharacterized LOC4337577	PREDICTED: LOW QUALITY PROTEIN: probable tRNA pseudouridine synthase 1 partial [Brachypodium distachyon]	avena_sativa_T52545	Pepsico2_Contig20777.path2
avena_sativa_T33623	0	6	104	0	0	0	2201	11.10394343	0.049996124	0.356277245	K08910|6.60205e-118|bdi:100830344|K08910 light-harvesting complex I chlorophyll a/b binding protein 4 | (RefSeq) chlorophyll a-b binding protein 4	Lhca4,chloroplastic PREDICTED: chlorophyll a-b binding protein 4	avena_sativa_T33623	Pepsico1_Contig8179.path1
avena_sativa_T32109	60	45	3	0	0	0	2161	11.07748336	0.00000347	0.000346175	NA		avena_sativa_T32109	TRINITY_DN74454_c0_g1_i1.path1
avena_sativa_T39457	39	10	57	0	0	0	2121	11.05052891	0.000000194	0.000031	K02927|2.9595e-86|bdi:100828943|K02927 large subunit ribosomal protein L40e | (RefSeq) ubiquitin-60S ribosomal protein L40-2	PREDICTED: ubiquitin-60S ribosomal protein L40-2 [Brachypodium distachyon]	avena_sativa_T39457	TRINITY_DN6478_c0_g1_i3.path1
avena_sativa_T43153	55	36	9	0	0	0	2001	10.96650545	0.000000354	0.0000509	--	unnamed protein product [Triticum aestivum]	avena_sativa_T43153	Pepsico2_Contig4722.path2
avena_sativa_T16471	30	31	37	0	0	0	1961	10.93737382	5.53E-09	0.00000143	--	hypothetical protein F775_43951 [Aegilops tauschii]	avena_sativa_T16471	TRINITY_DN526_c0_g1_i2.path1
avena_sativa_T6439	35	43	2	0	0	0	1601	10.64475759	0.0000204	0.001443419	K08912|1.69844e-168|bdi:100822184|K08912 light-harvesting complex II chlorophyll a/b binding protein 1 | (RefSeq) chlorophyll a-b binding protein of LHCII type 1	Lhcb1,PREDICTED: chlorophyll a-b binding protein of LHCII type 1 [Brachypodium distachyon]	avena_sativa_T6439	Pepsico1_Contig7367.path1
avena_sativa_T46855	21	16	30	0	0	0	1341	10.38909352	0.000000287	0.000042	--	hypothetical protein F775_29669 [Aegilops tauschii]	avena_sativa_T46855	Pepsico2_Contig13183.path1
avena_sativa_T302	20	22	23	0	0	0	1301	10.34540525	0.000000183	0.0000302	--	H0321H01.8 [Oryza sativa Indica Group]	avena_sativa_T302	Pepsico1_Contig38883.path1
avena_sativa_T6284	23	24	17	0	0	0	1281	10.32305476	0.000000262	0.0000394	NA		avena_sativa_T6284	Pepsico2_Contig9392.path1
avena_sativa_T11591	24	21	15	0	0	0	1201	10.23002044	0.000000525	0.0000721	NA		avena_sativa_T11591	NA
avena_sativa_T9938	8	12	39	0	0	0	1181	10.20579325	0.0000106	0.000874101	--	putative acyl transferase 4 [Triticum aestivum]	avena_sativa_T9938	Pepsico2_Contig5141.path2
avena_sativa_T35146	18	17	16	0	0	0	1021	9.995767151	0.00000136	0.00016379	K02503|2.24007e-74|bdi:100841085|K02503 histidine triad (HIT) family protein | (RefSeq) 14 kDa zinc-binding protein	predicted protein [Hordeum vulgare subsp. vulgare]	avena_sativa_T35146	Pepsico1_Contig1331.path2
avena_sativa_T42278	16	19	11	0	0	0	921	9.847057346	0.00000462	0.000434393	--	PREDICTED: uncharacterized protein LOC100828179 [Brachypodium distachyon]	avena_sativa_T42278	Pepsico1_Contig23543.path1
avena_sativa_T36826	14	18	13	0	0	0	901	9.815383296	0.00000426	0.000410896	NA		avena_sativa_T36826	NA
avena_sativa_T1057	13	16	14	0	0	0	861	9.749869427	0.00000544	0.000482924	K00864|0|bdi:100831505|K00864 glycerol kinase [EC:2.7.1.30] | (RefSeq) glycerol kinase	glycerol kinase [Triticum aestivum]	avena_sativa_T1057	Pepsico1_Contig30909.path1
avena_sativa_T39793	15	14	14	0	0	0	861	9.749869427	0.00000519	0.000470217	--	hypothetical protein F775_00011 [Aegilops tauschii]	avena_sativa_T39793	Pepsico1_Contig36560.path1
avena_sativa_T48897	14	7	21	0	0	0	841	9.71596199	0.0000224	0.001558007	K05359|6.9605e-103|bdi:100821339|K05359 arogenate/prephenate dehydratase [EC:4.2.1.91 4.2.1.51] | (RefSeq) arogenate dehydratase/prephenate dehydratase 6	chloroplastic-like predicted protein [Hordeum vulgare subsp. vulgare]	avena_sativa_T48897	Pepsico1_Contig23982.path1
avena_sativa_T59924	17	16	9	0	0	0	841	9.71596199	0.000011	0.000897087	K03363|2.29468e-19|bdi:100835337|K03363 cell division cycle 20	cofactor of APC complex | (RefSeq) cell division cycle 20.2 cofactor of APC complex-like	avena_sativa_T59924	TRINITY_DN11571_c0_g1_i4.path1
avena_sativa_T36793	11	10	20	0	0	0	821	9.681238412	0.0000153	0.001147087	K09874|1.75047e-123|bdi:100834694|K09874 aquaporin NIP | (RefSeq) aquaporin NIP2-2	silicon transporter [Hordeum vulgare]	avena_sativa_T36793	Pepsico1_Contig4641.path1
avena_sativa_T54682	0	2	39	0	0	0	821	9.681238412	0.009328315	0.135876876	NA		avena_sativa_T54682	NA

avena_sativa_T9687	13	18	9	0	0	0	801	9.645658432	0.0000159	0.001182585	NA		avena_sativa_T9687	NA
avena_sativa_T22998	19	16	4	0	0	0	781	9.609178738	0.0000869	0.004485239	--	retrotransposon protein putative	avena_sativa_T22998	TRINITY_DN18986_c0_g1_i3.path1
avena_sativa_T24133	14	11	14	0	0	0	781	9.609178738	0.000012	0.000960188	--	predicted protein [Hordeum vulgare subsp. vulgare]	avena_sativa_T24133	Pepsico2_Contig1328.path1
avena_sativa_T5556	0	2	36	0	0	0	761	9.571752644	0.010887443	0.150098408	--	unnamed protein product [Triticum aestivum]	avena_sativa_T5556	Pepsico1_Contig4433.path1
avena_sativa_T5436	10	12	13	0	0	0	701	9.453270634	0.0000263	NA	K08770|0|dosa:Os06t0681400-01|K08770 ubiquitin C | (RAP-DB) Os06g0681400; Ubiquitin domain containing protein.	PREDICTED: polyubiquitin [Eucalyptus grandis]	avena_sativa_T5436	Pepsico1_Contig5911.path1
avena_sativa_T51214	18	15	1	0	0	0	681	9.411510988	0.000980271	NA	K10258|0|bdi:100830422|K10258 very-long-chain enoyl-CoA reductase [EC:1.3.1.93] | (RefSeq) very-long-chain enoyl-CoA reductase	PREDICTED: very-long-chain enoyl-CoA reductase [Brachypodium distachyon]	avena_sativa_T51214	Pepsico2_Contig11401.path1
avena_sativa_T16765	9	11	13	0	0	0	661	9.368506462	0.000043	NA	--	Serine/threonine-phosphatase BSL2-like protein [Aegilops tauschii]	avena_sativa_T16765	Pepsico1_Contig30513.path1
avena_sativa_T46004	1	15	17	0	0	0	661	9.368506462	0.001080867	NA	K00281|0|bdi:100845191|K00281 glycine dehydrogenase [EC:1.4.4.2] | (RefSeq) glycine dehydrogenase (decarboxylating) 2	mitochondrial victorin binding protein [Avena sativa]	avena_sativa_T46004	Pepsico2_Contig16080.path1
avena_sativa_T52676	9	10	13	0	0	0	641	9.324180547	0.0000541	NA	K01115|3.1098e-17|bdi:100840776|K01115 phospholipase D1/2 [EC:3.1.4.4] | (RefSeq) phospholipase D delta	Phospholipase D delta [Triticum urartu]	avena_sativa_T52676	TRINITY_DN3598_c0_g1_i10.path1
avena_sativa_T59016	0	14	18	0	0	0	641	9.324180547	0.007007118	NA	NA		avena_sativa_T59016	Pepsico2_Contig9889.path2
avena_sativa_T7746	207	178	287	0	0	1	640.047619	9.322035434	4.5E-14	3.79E-11	NA		avena_sativa_T7746	TRINITY_DN8506_c1_g1_i1.path1
avena_sativa_T2602	9	10	11	0	0	0	601	9.231221181	0.0000763	NA	K07513|0|bdi:100834503|K07513 acetyl-CoA acyltransferase 1 [EC:2.3.1.16] | (RefSeq) 3-ketoacyl-CoA thiolase 2	peroxisomal 3-ketoacyl-CoA thiolase-like protein [Triticum aestivum]	avena_sativa_T2602	Pepsico2_Contig1480.path1
avena_sativa_T42613	14	15	1	0	0	0	601	9.231221181	0.001470632	NA	K07466|3.25707e-13|osa:4327373|K07466 replication factor A1 | (RefSeq) P0031D11.8; replication protein A 70 kDa DNA-binding subunit B-like	Os01g0838100 [Oryza sativa Japonica Group]	avena_sativa_T42613	Pepsico2_Contig11844.path1
avena_sativa_T5176	0	2	28	0	0	0	601	9.231221181	0.01745072	NA	K08770|0|bdi:100831055|K08770 ubiquitin C | (RefSeq) polyubiquitin	PREDICTED: polyubiquitin-like [Brachypodium distachyon]	avena_sativa_T5176	Pepsico2_Contig19302.path1
avena_sativa_T13430	13	8	8	0	0	0	581	9.182394353	0.000126727	NA	NA		avena_sativa_T13430	Pepsico2_Contig6181.path1
avena_sativa_T33594	11	8	10	0	0	0	581	9.182394353	0.00010297	NA	NA		avena_sativa_T33594	NA

* data from the website of https://wheat.pw.usda.gov/GG3/graingenes downloads/oat-ot3098-pepsico

Supplementary Table 2. Top 50 up/down-regulated DEGs during the oat bracts development.

Top 50 down-regulated DEGs at milk stage(TCm vs QHm)

Gene ID	L3a.1	L3a.2	L3a.3	L4a.1	L4a.2	L4a.3	L3a_L4a_fc	L3a_L4a_fc2	L3a_L4a_pvalue	L3a_L4a_padj	KEGG	Annotation	Challenger Gene ID	oat-ot3098-pepsico*
avena_sativa_T5324	0	374	1552	0	0	0	38521	15.23335754	0.002839257	0.201681409	NA		avena_sativa_T5324	Pepsico2_Contig5893.path1
avena_sativa_T6406	242	479	159	0	0	0	17601	14.10336978	6.65E-16	1.54E-12	K08910|7.97399e-153|bdi:100830344|K08910 light-harvesting complex I chlorophyll a/b binding protein 4 | (RefSeq) chlorophyll a-b binding protein 4	Lhca4,chloroplastic PREDICTED: chlorophyll a-b binding protein 4	avena_sativa_T6406	Pepsico1_Contig8179.path1
avena_sativa_T6255	165	214	372	0	0	0	15021	13.87469324	2.47E-16	6.25E-13	NA		avena_sativa_T6255	Pepsico2_Contig2416.path1
avena_sativa_T7965	305	148	181	0	0	0	12681	13.6303809	1.63E-15	3.5E-12	NA		avena_sativa_T7965	Pepsico1_Contig495.path1
avena_sativa_T5397	124	143	333	0	0	0	12001	13.550867	9.1E-15	1.71E-11	NA		avena_sativa_T5397	Pepsico1_Contig849.path2
avena_sativa_T45153	129	127	220	0	0	0	9521	13.21689739	9.2E-15	1.71E-11	--	PREDICTED: thioredoxin H-type-like [Brachypodium distachyon]	avena_sativa_T45153	Pepsico1_Contig1731.path1
avena_sativa_T20589	106	116	218	0	0	0	8801	13.10345174	4.15E-14	6.43E-11	--	predicted protein [Hordeum vulgare subsp. vulgare]	avena_sativa_T20589	Pepsico2_Contig11082.path1
avena_sativa_T6842	79	95	260	0	0	0	8681	13.08364553	6.15E-13	7.78E-10	NA		avena_sativa_T6842	Pepsico2_Contig18164.path1
avena_sativa_T54029	259	25	26	0	0	0	6201	12.59828517	7.57E-09	0.00000466	--	predicted protein [Hordeum vulgare subsp. vulgare]	avena_sativa_T54029	NA
avena_sativa_T33664	63	86	113	0	0	0	5241	12.35562639	2.84E-12	3.29E-09	K04078|1.59513e-138|bdi:100837856|K04078 chaperonin GroES | (RefSeq) 20 kDa chaperonin	chloroplastic PREDICTED: chlorophyll a-b binding protein 4	avena_sativa_T33664	Pepsico2_Contig3249.path1
avena_sativa_T9994	0	33	183	0	0	0	4321	12.07714952	0.029697313	0.807540699	--	predicted protein [Hordeum vulgare subsp. vulgare]	avena_sativa_T9994	Pepsico1_Contig1911.path1
avena_sativa_T49334	81	59	68	0	0	0	4161	12.02271457	1.63E-11	1.74E-08	--	hypothetical protein F775_29669 [Aegilops tauschii]	avena_sativa_T49334	Pepsico2_Contig13183.path1
avena_sativa_T54168	40	39	102	0	0	0	3621	11.82217246	5.52E-10	0.000000466	NA		avena_sativa_T54168	TRINITY_DN16761_c0_g1_i1.path1
avena_sativa_T33623	46	89	42	0	0	0	3541	11.78994113	3.95E-10	0.000000367	K08910|6.60205e-118|bdi:100830344|K08910 light-harvesting complex I chlorophyll a/b binding protein 4 | (RefSeq) chlorophyll a-b binding protein 4	Lhca4,chloroplastic PREDICTED: chlorophyll a-b binding protein 4	avena_sativa_T33623	Pepsico1_Contig8179.path1
avena_sativa_T39457	27	49	84	0	0	0	3201	11.64430696	1.52E-09	0.00000112	K02927|2.9595e-86|bdi:100828943|K02927 large subunit ribosomal protein L40e | (RefSeq) ubiquitin-60S ribosomal protein L40-	PREDICTED: ubiquitin-60S ribosomal protein L40-2 [Brachypodium distachyon]	avena_sativa_T39457	TRINITY_DN6478_c0_g1_i3.path1
avena_sativa_T7022	132	0	16	0	0	0	2961	11.53186878	0.039253616	0.91465853	--	PREDICTED: TATA-binding protein 2-like isoform X1 [Brachypodium distachyon]	avena_sativa_T7022	TRINITY_DN80635_c0_g1_i1.path1
avena_sativa_T6230	34	52	61	0	0	0	2941	11.52209107	6.52E-10	0.00000053	NA		avena_sativa_T6230	Pepsico2_Contig19793.path1
avena_sativa_T28896	93	22	29	0	0	0	2881	11.49235395	2.44E-08	0.0000128	--	unnamed protein product [Triticum aestivum]	avena_sativa_T28896	Pepsico2_Contig543.path1
avena_sativa_T42093	18	40	73	0	0	0	2621	11.35590164	0.000000018	0.00001	--	predicted protein [Hordeum vulgare subsp. vulgare]	avena_sativa_T42093	Pepsico2_Contig11082.path1
avena_sativa_T32109	31	44	54	0	0	0	2581	11.33371443	1.89E-09	0.00000135	NA		avena_sativa_T32109	TRINITY_DN74454_c0_g1_i1.path1
avena_sativa_T46855	36	30	46	0	0	0	2241	11.12992693	5.08E-09	0.00000329	--	hypothetical protein F775_29669 [Aegilops tauschii]	avena_sativa_T46855	Pepsico2_Contig13183.path1
avena_sativa_T60490	76	17	14	0	0	0	2141	11.06406908	0.000000585	0.000210853	K15404|5.97059e-103|bdi:100833295|K15404 aldehyde decarbonylase [EC:4.1.99.5] | (RefSeq) protein ECERIFERUM 1-like	PREDICTED: protein ECERIFERUM 1-like [Brachypodium distachyon]	avena_sativa_T60490	Pepsico2_Contig6620.path1
avena_sativa_T32107	24	56	26	0	0	0	2121	11.05052891	3.97E-08	0.0000201	--	hypothetical protein F775_32419 [Aegilops tauschii]	avena_sativa_T32107	Pepsico1_Contig4308.path1
avena_sativa_T20709	74	16	15	0	0	0	2101	11.03686045	0.000000591	0.000210853	K15404|0|bdi:100833807|K15404 aldehyde decarbonylase [EC:4.1.99.5] | (RefSeq) protein ECERIFERUM 1-like	PREDICTED: protein ECERIFERUM 1-like [Brachypodium distachyon]	avena_sativa_T20709	Pepsico2_Contig6620.path1
avena_sativa_T31258	28	42	33	0	0	0	2061	11.00912879	1.26E-08	0.00000716	NA		avena_sativa_T31258	TRINITY_DN72452_c0_g1_i1.path1
avena_sativa_T10314	80	1	2	0	0	0	1661	10.69783636	0.000361696	0.046412063	--	hypothetical protein TRIUR3_01588 [Triticum urartu]	avena_sativa_T10314	TRINITY_DN20164_c0_g1_i1.path1
avena_sativa_T53822	25	25	31	0	0	0	1621	10.66266838	7.36E-08	0.0000354	--	Myb family transcription factor APL [Aegilops tauschii]	avena_sativa_T53822	TRINITY_DN26742_c0_g2_i1.path1
avena_sativa_T64252	27	28	25	0	0	0	1601	10.64475759	8.36E-08	0.0000395	--	PREDICTED: uncharacterized protein LOC100846834 [Brachypodium distachyon]	avena_sativa_T64252	TRINITY_DN569_c0_g1_i11.path1
avena_sativa_T12141	21	27	27	0	0	0	1501	10.55170826	0.000000154	0.000067	K14457|6.0278e-33|obr:102707405|K14457 2-acylglycerol O-acyltransferase 2 [EC:2.3.1.22] | (RefSeq) diacylglycerol O-acyltransferase 2	unnamed protein product [Oryza sativa Japonica Group]	avena_sativa_T12141	NA
avena_sativa_T65971	18	27	30	0	0	0	1501	10.55170826	0.000000201	0.0000825	NA		avena_sativa_T65971	Pepsico1_Contig34402.path2
avena_sativa_T47566	35	15	21	0	0	0	1421	10.47269084	0.000000641	0.000223103	K15103|1.47142e-32|bdi:100834437|K15103 solute carrier family 25 (mitochondrial uncoupling protein)	member 8/9 | (RefSeq) mitochondrial uncoupling protein 1-like PREDICTED: mitochondrial uncoupling protein 1-like [Brachypodium distachyon]	avena_sativa_T47566	Pepsico2_Contig17691.path2

avena_sativa_T58021	17	19	32	0	0	0	1361	10.41045135	0.000000559	0.000204762	K10251|4.23541e-117|sbi:SORBI_01g047620|K10251 17beta-estradiol 17-dehydrogenase / very-long-chain 3-oxoacyl-CoA reductase [EC:1.1.1.62 1.1.1.330] | (RefSeq) SORBIDRAFT_01g047620	Sb01g047620; hypothetical protein predicted protein [Hordeum vulgare subsp. vulgare]	avena_sativa_T58021	Pepsico1_Contig5742.path1
avena_sativa_T50041	24	21	22	0	0	0	1341	10.38909352	0.00000037	0.000139139	--	hypothetical protein TRIUR3_20374 [Triticum urartu]	avena_sativa_T50041	NA
avena_sativa_T66151	25	15	19	0	0	0	1181	10.20579325	0.00000147	0.000442538	K17086|0|bdi:100839421|K17086 transmembrane 9 superfamily member 2/4 | (RefSeq) transmembrane 9 superfamily member 8-like	PREDICTED: transmembrane 9 superfamily member 4-like [Brachypodium distachyon]	avena_sativa_T66151	Pepsico1_Contig30645.path1
avena_sativa_T20972	13	11	28	0	0	0	1041	10.02375435	0.00000804	0.001881267	--	hypothetical protein F775_29669 [Aegilops tauschii]	avena_sativa_T20972	Pepsico2_Contig13183.path1
avena_sativa_T46282	16	18	18	0	0	0	1041	10.02375435	0.0000029	0.000792943	NA		avena_sativa_T46282	TRINITY_DN197_c1_g1_i2.path1
avena_sativa_T11591	14	14	21	0	0	0	981	9.938109326	0.00000558	0.001374571	NA		avena_sativa_T11591	NA
avena_sativa_T13006	15	19	15	0	0	0	981	9.938109326	0.00000515	0.00130645	K09286|1.47097e-96|bdi:100836054|K09286 EREBP-like factor | (RefSeq) ethylene-responsive transcription factor RAP2-13	PREDICTED: ethylene-responsive transcription factor RAP2-13-like [Brachypodium distachyon]	avena_sativa_T13006	TRINITY_DN4150_c1_g1_i1.path1
avena_sativa_T32165	16	12	21	0	0	0	981	9.938109326	0.00000608	0.001473329	K12403|2.41954e-70|bdi:100834030|K12403 AP-4 complex subunit sigma-1 | (RefSeq) AP-4 complex subunit sigma	AP-4 complex subunit sigma [Triticum urartu]	avena_sativa_T32165	TRINITY_DN2073_c0_g1_i1.path1
avena_sativa_T36826	15	17	13	0	0	0	901	9.815383296	0.0000099	0.00227906	NA		avena_sativa_T36826	NA
avena_sativa_T51118	11	25	8	0	0	0	881	9.782998209	0.0000392	0.007272332	NA		avena_sativa_T51118	TRINITY_DN89657_c0_g1_i1.path1
avena_sativa_T7031	11	9	24	0	0	0	881	9.782998209	0.0000279	0.005501096	K07904|3.13725e-133|bdi:100836026|K07904 Ras-related protein Rab-11A | (RefSeq) ras-related protein RABA2a-like	predicted protein [Hordeum vulgare subsp. vulgare]	avena_sativa_T7031	Pepsico2_Contig8998.path1
avena_sativa_T13056	9	10	24	0	0	0	861	9.749869427	0.0000354	0.006753659	K11096|6.95213e-63|osa:4338385|K11096 small nuclear ribonucleoprotein D2 | (RefSeq) small nuclear ribonucleoprotein Sm D2	Os05g0314100 [Oryza sativa Japonica Group]	avena_sativa_T13056	Pepsico1_Contig1465.path1
avena_sativa_T51960	12	11	17	0	0	0	801	9.645658432	0.0000254	0.005166967	NA		avena_sativa_T51960	TRINITY_DN48095_c0_g1_i2.path1
avena_sativa_T18387	13	13	12	0	0	0	761	9.571752644	0.0000319	0.006133768	NA		avena_sativa_T18387	TRINITY_DN14716_c0_g2_i2.path1
avena_sativa_T36793	11	16	11	0	0	0	761	9.571752644	0.0000385	0.007195277	K09874|1.75047e-123|bdi:100834694|K09874 aquaporin NIP | (RefSeq) aquaporin NIP2-2	silicon transporter [Hordeum vulgare]	avena_sativa_T36793	Pepsico1_Contig4641.path1
avena_sativa_T16471	37	0	0	0	0	0	741	9.533329732	0.032255093	0.843717593	--	hypothetical protein F775_43951 [Aegilops tauschii]	avena_sativa_T16471	TRINITY_DN526_c0_g1_i2.path1
avena_sativa_T1679	14	10	13	0	0	0	741	9.533329732	0.000042	0.007639918	K01412|0|bdi:100822433|K01412 mitochondrial-processing peptidase subunit alpha [EC:3.4.24.64] | (RefSeq) mitochondrial-processing peptidase subunit alpha-like	PREDICTED: mitochondrial-processing peptidase subunit alpha-like [Brachypodium distachyon]	avena_sativa_T1679	Pepsico1_Contig20344.path1
avena_sativa_T41981	11	10	16	0	0	0	741	9.533329732	0.0000459	0.008241338	K03347|2.32031e-136|bdi:100824990|K03347 cullin 1 | (RefSeq) cullin-1-like	Cullin-1 [Aegilops tauschii]	avena_sativa_T41981	Pepsico2_Contig10425.path2
avena_sativa_T48103	9	10	18	0	0	0	741	9.533329732	0.0000596	0.010313203	NA		avena_sativa_T48103	Pepsico2_Contig4906.path1

* data from the website of https://wheat.pw.usda.gov/GG3/graingenes_downloads/oat-ot3098-pepsico

Supplementary Table 2. Top 50 up/down-regulated DEGs during the oat bracts development.
